# Transcriptomic landscape of pseudorabies virus-induced encephalitis reveals key lncRNAs involved in host–neurotropic virus interactions

**DOI:** 10.1186/s13567-025-01650-5

**Published:** 2025-11-10

**Authors:** Thach Phan Van, Tien Huyen Ton Nu Bao, Byungkwan Oh, Ngan Tran Thi Kim, Sang-Ik Oh, Bumseok Kim

**Affiliations:** 1 Laboratory of Veterinary Pathology and Biosafety Research Institute, College of Veterinary Medicine, Jeonbuk National University, Iksan, 54596 Republic of Korea; 2 Faculty of Applied Science and Technology (FAST), Nguyen Tat Thanh University, Ho Chi Minh City, 700000 Vietnam; 3 Department of Husbandry and Veterinary Medicine, School of Agriculture and Natural Resources, Vinh University, 182 Le Duan, Vinh, Nghe An, 460000 Vietnam

**Keywords:** Pseudorabies virus, encephalitis, long noncoding RNA, immune response, RNA sequencing

## Abstract

Pseudorabies virus (PRV) infection causes fatal encephalitis across various species, a condition known as pseudorabies encephalitis (PRE). However, the molecular mechanisms underlying PRE remain poorly understood. Long noncoding RNAs (lncRNAs) have emerged as important regulators of gene expression in neurological diseases and viral infections. This study explores genome-wide transcriptional alterations in a mouse model of PRV-induced encephalitis. The intranasal inoculation of mice with PRV induced severe encephalitis, characterized by high viral loads, significant inflammatory responses, and the onset of neurological symptoms. RNA–seq analysis revealed 683 differentially expressed (DE) mRNAs and 179 DElncRNAs in PRV-infected brains compared with controls. Functional and pathway analyses revealed that PRE involves neurodegeneration and diverse immune responses, reflecting the complex interplay between viral evasion strategies and host defenses. Co-expression network analysis, supported by experimental validation, identified several lncRNAs as central hubs interacting with multiple immune-related genes and exhibiting cell type-specific expression pattern. Notably, ZFAS1 was prominently dysregulated in PRV-infected microglia and showed extensive co-expression connectivity. Knockdown of ZFAS1 modulated microglia-driven inflammation without altering viral replication, underscoring its potential as a therapeutic target for mitigating neuroinflammation in virus-associated neurological diseases. The identification of key lncRNAs and their potential regulatory roles deepens our understanding of disease mechanisms, while offering new avenues for therapeutic intervention in neurotropic viral infections.

**Supplementary Information:**

The online version contains supplementary material available at 10.1186/s13567-025-01650-5.

## Introduction

Pseudorabies virus (PRV), or Suid herpesvirus 1, is a neurotropic alpha herpesvirus that primarily infects swine, causing Aujeszky’s disease, a highly contagious and economically significant disease in the livestock industry [1]. Beyond swine, PRV infects a broad range of hosts, including cattle, rodent, carnivore, and human, where it can cause fatal encephalitis [2, 3]. Pseudorabies encephalitis (PRE) is characterized by severe neurological symptoms that include seizures, paralysis, and respiratory distress, often leading to death [4, 5]. In human, PRE cases are sporadic, with recent outbreaks linked to emerging PRV genotype II strains that exhibit enhanced neurovirulence and zoonotic potential [3, 5, 6]. Clinical observations have shown that all infected individuals present with profound neurological symptoms, with a 16% case fatality rate, while 95% of survivors experience severe neurological sequelae [7]. These strains have raised concerns about the public health implications of PRV infections, particularly in rural areas with close human–animal contact.

The neuropathology of PRE involves rapid viral replication in the central nervous system (CNS), leading to robust neuroinflammation and neuronal damage [3, 8]. Although studies have explored immune responses to PRV, the molecular mechanisms of its transcriptional regulation during infection remain unclear. Previous transcriptomic studies in rodent and pig, using microarray-based technology, revealed changes in immune and stress-related genes, but were limited by resolution and the absence of species-specific probes [9, 10]. These studies focused mainly on abundant coding RNAs, neglecting lncRNAs, which are emerging as key regulators of immune responses and neuronal survival during viral infections.

Advances in transcriptomic technologies enable genome-wide analyses of host responses to viral infections, offering valuable insights into the complex interplay between viruses and host cells [11–13]. Long noncoding RNAs (lncRNAs), a class of regulatory RNAs with diverse roles in gene expression, have emerged as critical mediators of host–pathogen interactions [14, 15]. Recent studies have highlighted the involvement of lncRNAs in modulating immune responses, neuronal survival, and inflammation during viral infections, including herpes simplex virus (HSV) and Japanese encephalitis virus (JEV) infections [16, 17]. Fang et al. identified lncRNA LNC_000641 as a regulator of PRV replication in alveolar macrophages, suggesting that it could be a potential target for antiviral therapies against PRV infection [18]. Given the emerging role of lncRNAs in regulating viral infections and neuroinflammation, a comprehensive analysis of mRNA and lncRNA expression profiles during PRV-induced encephalitis is warranted.

To address this knowledge gap, we performed a comprehensive transcriptomic analysis of mouse brains following intranasal PRV infection, focusing on both mRNA and lncRNA expression profiles. By integrating differential expression analysis, functional enrichment, co-expression network construction, and experimental validation, we aimed to identify key regulatory lncRNAs involved in PRV-induced neuropathology. Our findings provide novel insights into the molecular landscape of PRV neuropathogenesis and underscore the pivotal role of lncRNAs in orchestrating host immune and neuroinflammatory responses. This study lays the groundwork for future investigations into lncRNA-based diagnostics and therapeutics targeting neurotropic viral infections.

## Materials and methods

### Viral strain and animal experimental design

The PRV Yangsan (YS) viral strain used in this study was preserved by the College of Veterinary Medicine and Bio-Safety Research Institute, Jeonbuk National University (Iksan, Korea), and propagated in PK-15 cells. Each virus stock was titrated by determining the TCID₅₀ using the Reed–Muench method on PK-15 cells.

Male C57BL/6 J mice, of 6–7 weeks’ age, were purchased from Samtako Bio-Korea (Osan, Republic of Korea), and maintained under standard conditions at the animal facility at Jeonbuk National University according to the Institutional Guidelines. For intranasal inoculation, mice were anesthetized with Zoletil (tiletamine-zolazepam, Virbac, Carros, France) at a dose of 0.2 mL/kg body weight through intramuscular injection prior to the administration of 20 µL of medium containing 1 × 10^5^ TCID₅₀ virus, which was delivered as 10 µL per nostril. To monitor survival rates, body weight changes, and clinical symptoms, five mice each from the control and infected groups were observed twice daily. For RNA sequencing (RNA–seq), RT‒qPCR, and immunohistochemistry (IHC), four mice from both the PRV-infected group and control group were euthanized and necropsied at 3 days post-inoculation (dpi). Preceding necropsy, the mice were anaesthetized with Zoletil at a dose of 0.2 mL/kg body weight administered intramuscularly. The whole brain was carefully isolated from each mouse. The left hemisphere of the brain was fixed in formalin for immunohistochemistry (IHC), while the right hemisphere was stored at −80 °C for subsequent molecular analyses, including RNA sequencing (RNA–seq) and RT‒qPCR. To assess viral burden in the brain, four PRV‒infected mice were anesthetized with Zoletil (0.2 mL/kg body weight, intramuscularly) and sacrificed at 3 days post-infection (dpi). Brain tissues were collected and homogenized in 2 mL of DMEM (Corning, USA) supplemented with 1% antibiotic–antimycotic using a Precellys Evolution homogenizer (Bertin Technologies, France). The homogenates were centrifuged to separate the pellet from the supernatant. Serial dilutions of the supernatants were used to infect PK-15 cells in 96-well plates, and virus titers were determined by calculating the 50% tissue culture infectious dose (TCID_50_) using the Reed–Muench method, expressed as log_10_TCID_50_/g.

### Cell culture and PRV infection

BV2 microglial cells were cultured in DMEM/F12 medium (Corning, USA) supplemented with 10% fetal bovine serum (FBS; Thermo Fisher Scientific Inc.) and 1% antibiotic–antimycotic solution (Thermo Fisher Scientific Inc.). N2a neuronal cells were maintained under the same incubation conditions in DMEM (Corning, USA) containing 10% FBS and 1% antibiotic–antimycotic. Both BV2 and N2a cells were seeded in 24-well plates and incubated at 37 °C in a humidified atmosphere with 5% CO_2_. Cells were infected with PRV at multiplicities of infection (MOIs) of 0.5 and 2. Following a 1-h adsorption period at 37 °C, the viral inoculum was removed and replaced with serum-free medium containing 1% antibiotic–antimycotic. Infected and mock-infected cells were harvested at 6- and 12-h post-infection (hpi) for RNA extraction.

### siRNA transfection

BV2 cells were transfected with siRNAs using Lipofectamine^™^ 2000 (Invitrogen), following the manufacturer’s protocol. After 24 h of transfection, cells were infected with PRV at a MOI of 2. Following a 1-h adsorption period at 37 °C, the viral inoculum was removed and replaced with serum-free medium containing 1% antibiotic–antimycotic. At 12 h post-infection, the cells were harvested for RT‒qPCR analysis, and the cell culture supernatant was collected for viral titration using the TCID_50_ assay. The siZFAS1 and negative control siRNA (siNC) oligonucleotides were synthesized by Bioneer Corporation (Daejeon, Korea).

### Immunohistochemistry

The samples collected and fixed were routinely processed and embedded in paraffin wax (Surgipath Paraplast, Leica Biosystems Inc., IL, USA). Formalin-fixed paraffin-embedded (FFPE) tissue blocks were sliced into 4 μm thick sections using a standard rotary microtome (HM–340E, Thermo Fisher Scientific Inc., MA, USA). To restore immunoreactivity, antigen retrieval was performed using citrate buffer (pH 6.0) at 95 °C for 30 min, followed by incubation at room temperature for 20 min. Slides were blocked by Super block (ScyTek Laboratories, Logan, UT) for 30 min. Separate sections were subsequently incubated overnight at 4 °C with rabbit anti-PRV antibody (PA1-081, Thermo Fisher Scientific) or rabbit anti-Iba1 antibody (A19776, ABclonal) diluted in antibody diluent (E09–300, GBI Labs, WA, USA). Detection of Iba1 labeling was achieved using ImmPRESS HRP Universal Antibody horseradish peroxidase-conjugated Anti-Mouse/Rabbit IgG antibody (MP–7500, Vector Laboratory, CA, USA). Visualization of the antibody was carried out with 3,3′–diaminobenzidine (DAB, SK–4105, Vector Laboratory, CA, USA), according to the manufacturer’s recommended concentration. Tissues were counterstained with hematoxylin. All slides were microscopically examined, and images were captured via a light microscope (BX53, Olympus, Tokyo, Japan) equipped with a camera (DP80, Olympus, Tokyo, Japan). Histopathological evaluations were conducted by trained pathologists in a double-blind manner. The quantification of the number of Iba1–positive (Iba1 +) cells in the control and infected groups was expressed as fold change.

### RNA isolation

The whole right mouse brains were homogenized using TRIzol reagent. Following homogenization, total RNA was extracted using the AccuPrep Universal RNA Extraction Kit (Bioneer, Korea). The isolated RNA was then dissolved in nuclease-free water, and stored at −80 °C, until further analysis. RNA purity and concentration were measured using a NanoDrop 2000 spectrophotometer (Thermo Scientific, USA).

### Reverse transcription quantitative PCR (RT‒qPCR)

The RNA was reverse transcribed into cDNA using ReverTra Ace^®^ qPCR RT Master Mix with gDNA Remover (Toyobo, Japan). Real-time PCR was performed using qPCRBIO SyGreen Blue Mix (PCR Biosystems, London, UK) on a CFX Opus 96 system (Bio-Rad, Singapore). After completion of the PCR cycles, specificity of the amplified products was confirmed by melting curve analysis. Gene expression levels were quantified using the comparative Ct method, with the Ct value of each sample normalized to that of the reference gene β–actin. Additional file 1 lists the sequences of primers used for PCR.

### Library preparation and sequencing

Total RNA was extracted, and its quality was assessed using an Agilent 2100 Bioanalyzer (Agilent Technologies, Palo Alto, CA, USA). All samples exhibited RNA integrity number (RIN) > 7 (Additional file 2), indicating high-quality RNA suitable for transcriptomic analysis. The TruSeq Stranded Total RNA with Ribo-Zero Gold kit (Illumina) was used for library preparation to retain both polyadenylated and nonpolyadenylated RNA species by depleting cytoplasmic and mitochondrial rRNAs. Sequencing was performed on the Illumina NovaSeq X platform (Illumina, San Diego, CA, USA) with paired-end reads (101 bp) by Macrogen (Seoul, Korea). High base quality was confirmed, with Q20 scores ranging from 97.9% to 98.3% and Q30 scores from 94.6% to 95.4% across all samples (Additional file 2), providing high-confidence data for downstream transcriptomic analyses.

### RNA–seq data preprocessing

The raw sequencing reads in FASTQ format were subjected to quality control using FastQC (v0.11.8) to assess read quality. Low-quality bases were trimmed using Sickle (version 1.33). Paired-end reads were processed to remove low-quality segments, while retaining high-confidence reads for downstream analysis. The trimmed reads were then aligned to the mouse reference genome (GRCm39) using STAR (2.7.11a). The gene expression levels were quantified with featureCounts (v1.6.0) using the GENCODE vM35 annotation file to count reads mapped. The resulting count matrix was used for subsequent differential expression analysis.

### Transcriptome analysis

#### Data import and gene type categorization

Raw RNA-sequencing count data were imported from the featureCounts output. Low-expression genes (those with counts ≤ 1 in fewer than two samples) were filtered out to improve downstream analysis reliability. Gene names and types were annotated according to the genome annotation files, and then categorized into broader functional groups: mRNA, long noncoding RNA (lncRNA), pseudogenes, microRNA (miRNA), other noncoding RNA, and transcripts of uncertain classification (TEC). Ambiguous gene type annotations were manually reviewed and corrected on the basis of external databases (MGI database, NCBI).

#### Sample clustering analysis

Principal component analysis (PCA): PCA plots were generated separately for mRNAs and lncRNAs to assess sample clustering. Euclidean Distance Heatmaps: Heatmaps visualizing sample-to-sample Euclidean distances were created for both the mRNA and lncRNA datasets.

#### Differential expression analysis

Differential expression analysis was performed using *DESeq2* package in the R programming language. Variance-stabilizing transformation (VST) was applied to normalize count data for visualization purposes. Differentially expressed genes (DEGs) were identified based on thresholds of adjusted *P* value < 0.05 (Benjamini‒Hochberg correction) and absolute log_2_ fold change > 1. Volcano plots highlighted significantly upregulated and downregulated genes separately for mRNA and lncRNA categories. Expression heatmaps of significant DEmRNAs and DElncRNAs were generated for the top 20 most significantly upregulated and downregulated genes.

#### Functional enrichment analysis

Gene Ontology (GO) enrichment analysis was performed using the *clusterProfiler* package in R to identify significantly enriched biological processes (BP) and molecular functions (MF) among DEmRNAs. Gene symbols were converted to Entrez IDs using the mouse annotation database (org.Mm.eg.db), and enrichment significance was determined using the Benjamini‒Hochberg method, with thresholds set to an adjusted *P* value < 0.05. Results were visualized using dot plots, highlighting the top 20 enriched GO terms.

In addition, gene set enrichment analysis (GSEA) was conducted using all expressed mRNA genes ranked by log_2_ fold-change values. GSEA was separately performed for GO biological process (BF) and molecular function (MF) categories, again employing *clusterProfiler* with Entrez ID annotations. Enriched gene sets were considered significant based on normalized enrichment scores (NES) and adjusted *P* value < 0.05. Visualization of significant gene sets was achieved by ridge plots, showcasing the top 20 terms ranked by NES for both BP and MF categories.

#### Pathway enrichment analysis

To provide clear visualization and biological interpretation of pathway level alterations, functional enrichment analysis was performed using Kyoto Encyclopedia of Genes and Genomes (KEGG) pathway data with the R packages *gage* and *pathview*. Gene set enrichment was conducted by ranking genes based on their log₂ fold changes. The *gage* package identified significantly enriched KEGG pathways associated with differential expression. The top pathways were visualized using the *pathview* package, which mapped significant gene expression changes onto KEGG pathway diagrams specific to mice (species code “mmu”).

#### Construction of a lncRNA‒mRNA co‒expression network

Differentially expressed mRNAs (DEmRNAs) and long noncoding RNAs (DElncRNAs) identified from RNA–seq analysis were used to construct a co-expression network. Significant correlations were defined as Pearson’s correlation coefficients greater than 0.99 or less than −0.99, with *P* values < 0.05. The top 1000 significant lncRNA‒mRNA pairs were selected on the basis of absolute correlation strength, and exported as an edge list for visualization [19]. The resulting network was exported in GraphML format, and visualized using Cytoscape software version 3.10.3. To concentrate on the co-regulatory network involving lncRNAs, we retained only those lncRNA hubs connected by two or more edges. Functional enrichment analysis of the modules was performed using the R package *clusterProfiler*.

### Data analysis and visualization

Transcriptomic data were analyzed and plotted in RStudio version 2024.12.1, using the above-mentioned R packages. Statistical analysis and graph generation for viral RNA copy number, fold change of Iba1 + cells, and relative mRNA and lncRNA expression were performed using Prism 8 (GraphPad Software). Statistical analysis was performed using unpaired *t* test and multiple *t* tests. Multiple *t* tests were corrected for multiple comparisons using the Holm‒Sidak method. All data are presented as mean ± SD. The differences were considered significant when *P* < 0.05.

## Results

### Establishment and characteristics of the PRE mouse model

**Figure 1:**
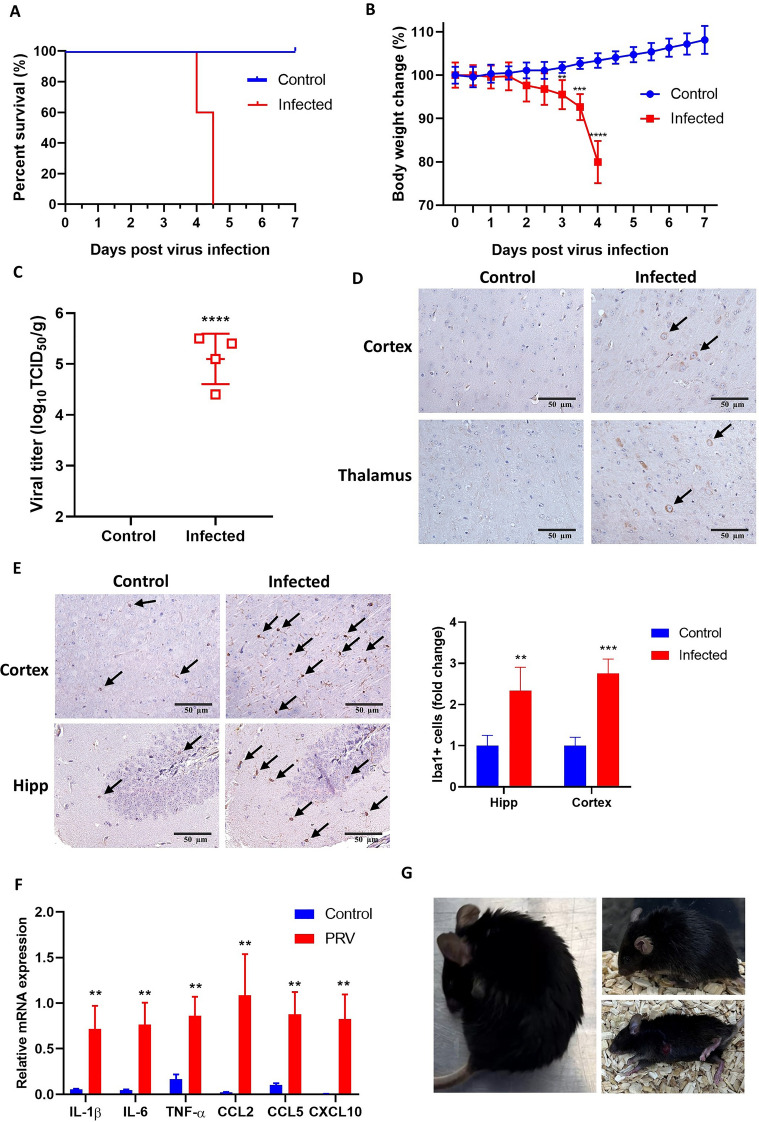
**Intranasal PRV infection induces PRE in mouse models.** **A** Kaplan‒Meier survival curves showing the survival rates of control and PRV-infected mice over time. **B** Body weight changes (%) in control and infected mice during infection. **C** TCID_50_ detection of virus titer in PRV-infected mouse brain (unpaired *t* test, *n* = 4). **D** Immunohistochemistry (IHC) staining for PRV antigens in brain sections showing PRV-positive neurons (arrows) in the cortex and thalamus. **E** IHC staining for Iba1 showing myeloid cells (arrows) in cortex and hippocampus (Hipp) of control and infected mice, with quantitative comparison (multiple *t* tests, *n* = 4). **F** Relative mRNA expression levels of proinflammatory factors (IL–1β, IL–6, TNF–**α**, CCL2, CCL5, and CXCL10) in brain tissues. *β*–actin was used as the internal control gene, and four biological replicates were used (multiple *t* tests, *n* = 4). The results are presented as the mean ± SD; *P < 0.05, **P < 0.01, ***P < 0.001, and ****P < 0.0001, compared with the control group. **G** Representative images of symptoms observed in PRV-infected mice, including a hunched posture with circling behavior (left), matted fur (upper right), and scratching accompanied by seizures (lower right).

Intranasal infection with PRV induced severe disease progression in mice, characterized by rapid weight loss, neurological symptoms, and high mortality. Survival analysis showed that all infected mice succumbed to the infection by 5 days post-infection (dpi), while control mice remained unaffected (Figure 1A). Infected mice experienced significant weight loss starting at 3 dpi, with a progressive decline until death, in contrast to the stable weight of control mice (Figure 1B).

Quantitative analysis at 3 dpi revealed a substantial viral burden, with viral titers of 4.4–5.5 log_10_ TCID_50_/g detected in brain tissues of infected mice (Figure 1C). Immunohistochemical staining for PRV antigens confirmed the presence of infected cells in the cortex and thalamus (Figure 1D). These findings indicate the efficiency of intranasal PRV injection in mimicking natural infection and driving robust viral replication in the brain. Infected brains also exhibited a strong neuroinflammatory response. IHC analysis for Iba1 showed marked myeloid cell infiltration in the hippocampus and cortex of PRV-infected mice compared with controls (Figure 1E). Correspondingly, mRNA expression levels of proinflammatory cytokines and chemokines—including IL–1β, IL–6, TNF–α, CCL2, CCL5, and CXCL10—were significantly elevated in infected brain tissues (Figure 1F), reflecting heightened immune activation.

PRV-infected mice developed progressive neurological symptoms starting at 3 dpi. Early signs included matting fur and hunched posture, progressing to scratching, convulsive movements, circling behavior, seizures, loss of balance, and eventual paralysis or death (Figure 1G, Additional file 3). These findings highlight the severe neurotropism and inflammatory response induced by PRV infection, leading to fatal encephalitis in this mouse model.

### PRV infection status determines characteristic mRNA and lncRNA expression patterns

**Figure 2:**
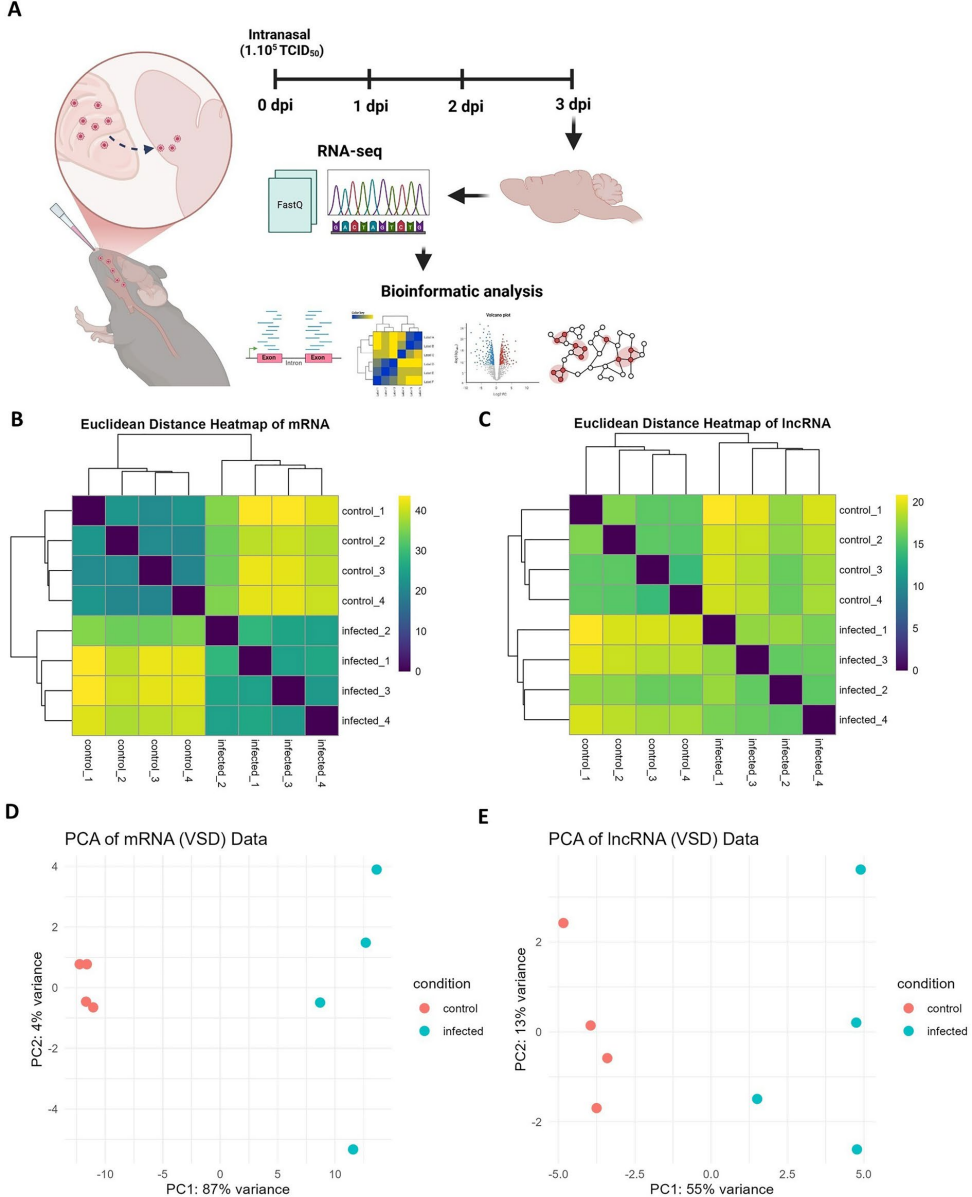
**PRV infection status determines characteristic transcriptomic profiles**. **A** Schematic of the transcriptomic profiling workflow for pseudorabies encephalitis. The diagram was constructed with BioRender. Heatmaps of Euclidean distances between samples based on **B** mRNA and **C** lncRNA profiles. Principal component analysis (PCA) of **D** mRNA and **E** lncRNA profiles across samples.

Transcriptomic profiling revealed that PRV infection induced significant alteration in both mRNA and lncRNA expression profiles, distinguishing infected samples from controls. Figure 2A presents a schematic of the experimental design.

Transcriptomic analysis revealed a diverse distribution of RNA species in the mouse brain. Among the 36,362 genes detected, mRNAs were the most abundant (18,572), followed by lncRNAs (7918), pseudogenes (5216), experimentally confirmed genes (TEC; 2668), other non‒coding RNAs (1313), and miRNAs (675) (Additional files 4A and B). Chromosomal analysis revealed that both mRNAs and lncRNAs were most abundant on chromosomes 2 and 7, and least abundant on chromosomes Y and mitochondrial chromosome (chrM), highlighting chromosomal variability in RNA distribution during PRV infection (Additional files 4C and D). These findings highlight the complexity of transcriptional regulation during PRV infection.

Heatmap analysis of Euclidean distances showed clear clustering of samples into two distinct groups: control and infected, for both mRNA and lncRNA profiles (Figures 2B, C). This clustering reflects the profound transcriptional changes associated with PRV infection. Principal component analysis (PCA) further confirmed this separation, with PC1 explaining 87% of the variance, and PC2 explaining 4% of the variance for mRNA profiles (Figure 2D). Similarly, PCA of lncRNA profiles revealed that PC1 accounted for 55% of the variance, while PC2 accounted for 13% (Figure 2E). These results highlight a strong transcriptional response to PRV infection, including both coding and noncoding RNA expression changes.

### Differential expression profiles of mRNAs and lncRNAs by RNA sequencing

**Figure 3:**
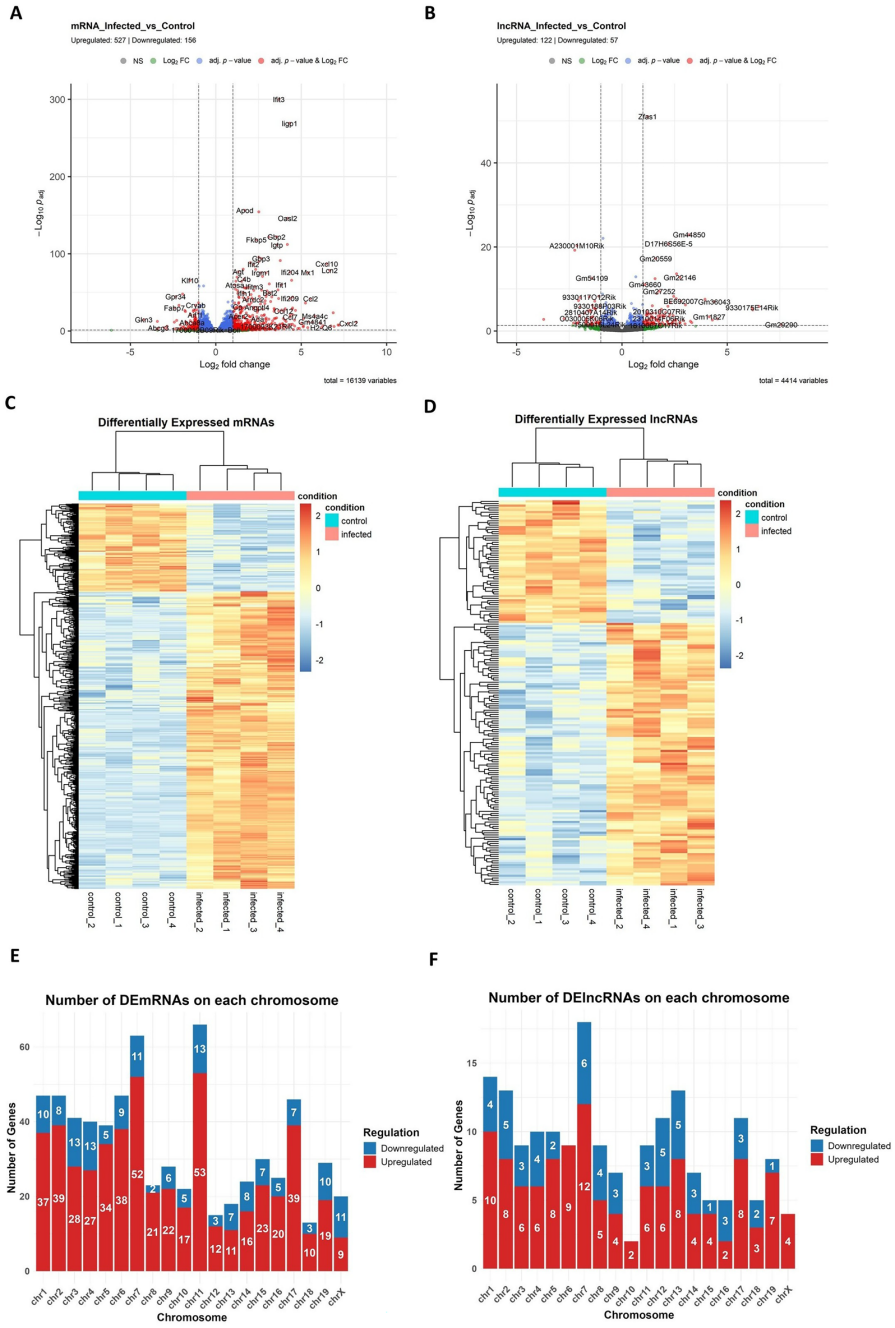
**Differential expression profiles of mRNAs and lncRNAs**. Volcano plot showing **A** DEmRNAs and **B** DElncRNAs, with adjusted *P* value < 0.05 and absolute log_2_ fold change > 1. Heatmap of **C** DEmRNA and **D** DElncRNA expression profiles in control and infected samples. Red through blue indicate high to low expression levels, respectively. Each column represents one sample, while each row corresponds with transcript. Bar chart shows the number of upregulated and downregulated **E** DEmRNAs and **F** DElncRNAs on each chromosome.

RNA sequencing analysis revealed significant changes in mRNA and lncRNA expression in the brains of PRV-infected mice, compared with those of control mice. Differentially expressed genes (DEGs) were identified based on an absolute log_2_-fold change greater than 1, and an adjusted *P* value of less than 0.05. Volcano plots show the distribution of DEmRNAs and DElncRNAs. A total of 527 mRNAs were upregulated, while 156 were downregulated (Figure 3A). Similarly, 122 lncRNAs were upregulated, while 57 were downregulated (Figure 3B). Heatmaps of DEmRNAs and DElncRNAs showed distinct expression patterns between the infected and control groups, further supporting the robust transcriptional changes induced by PRV infection (Figures 3C, D).

The chromosomal distribution of DEmRNAs and DElncRNAs was analyzed to determine their genomic localization. DEmRNAs were distributed across all chromosomes, with the highest numbers on chromosomes 7 and 11 ((63 and 66) DEmRNAs, respectively; Figure 3E). Similarly, DElncRNAs were also distributed across multiple chromosomes, with notable enrichment on chromosomes 1 and 7 ((14 and 18) DElncRNAs, respectively; Figure 3F). This widespread distribution suggests that PRV infection influences transcriptional activity throughout the genome.

### Validation of dysregulated mRNAs and lncRNAs

**Figure 4:**
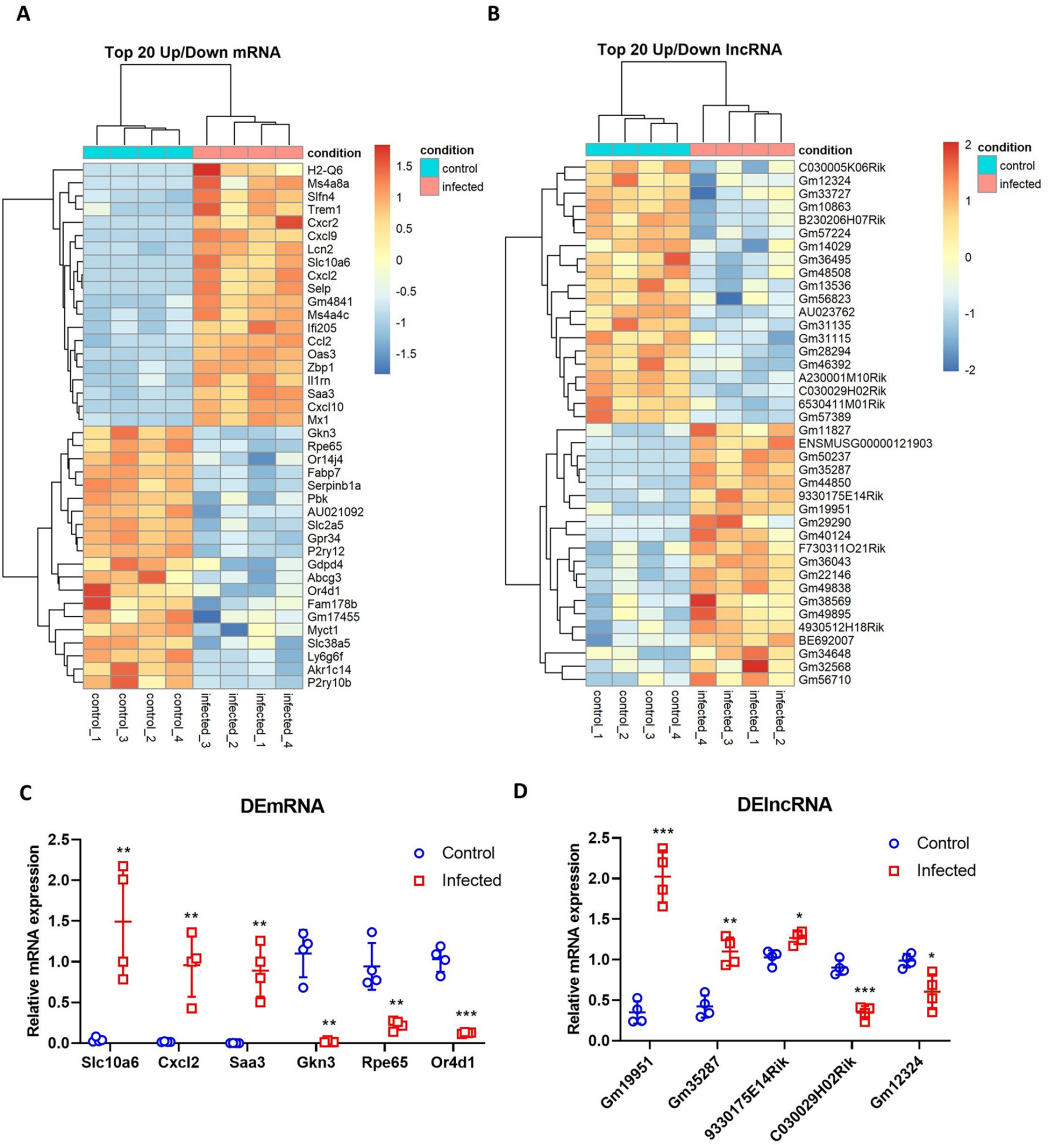
**Heatmaps and RT‒qPCR validation of the top differentially expressed genes in PRV-infected mouse brains**. Heatmap showing the top 20 upregulated and downregulated **A** DEmRNAs and **B** DElncRNAs in PRV-infected mice, compared with control mice. Red indicates upregulation, blue indicates downregulation, and clustering distinguishes samples. RT‒qPCR validation of selected **C** DEmRNAs and **D** DElncRNAs. Relative mRNA expression levels are shown for control (blue dots) and infected (red dots) samples. *β*–actin was used as the internal control gene, and four biological replicates were used. The results are presented as the mean ± SD; *P < 0.05, **P < 0.01, and ***P < 0.001, compared with the control group (multiple *t* tests, *n* = 4).

Transcriptomic analysis of the top 20 DEmRNAs and DElncRNAs showed distinct expression patterns between PRV-infected and control mice, as visualized in heatmaps (Figures 4A, B) and Additional files 5 and 6. Among the DEmRNAs, immune-related genes—including chemokines (Cxcl2, Cxcl9, Ccl2), immune receptors (Cxcr2, Trem1), and interferon-stimulated antiviral genes (Mx1, Oas3, Ifi205)—were predominantly upregulated, indicating an active immune response to PRV infection. Conversely, the expressions of neuronal-related genes (Fabp7), metabolism- and transport-related genes (Abcg3, Akr1c14), vision-related genes (Rpe65), and olfactory receptors (Or4d1, Or14j4) were downregulated. These findings suggest the suppression of neuronal function, metabolic processes, and sensory perception during PRV infection, likely reflecting a shift in cellular priorities toward immune defense at the expense of normal physiological functions. The top upregulated and downregulated DElncRNAs in the mouse brain following PRV-induced encephalitis were identified. The most significantly upregulated DElncRNAs were Gm19951, Gm50237, Gm35287, 9330175E14Rik, Gm11827, Gm40124, and Gm36043. Conversely, the top downregulated DElncRNAs were Gm46392, Gm31135, Gm12324, C030029H02Rik, Gm14029, and A230001M10Rik. These DElncRNAs exhibited distinct expression patterns in response to PRV infection in the mouse brain.

To validate the RNA–seq findings, RT‒qPCR analysis was performed on selected DEmRNAs and DElncRNAs (Figures 4C, D). Upregulated mRNAs (Cxcl2, Saa3, Slc10a6) exhibited significant increases (*P* < 0.01) in PRV-infected mice compared with controls, whereas downregulated mRNAs (Gkn3, Rpe65, Or4d1) showed marked reductions (*P* < 0.01). Similarly, RT‒qPCR confirmed the upregulation of lncRNAs (Gm19951, Gm35287, 9330175E14Rik) and the downregulation of lncRNAs (C030029H02Rik, Gm12324), with high statistical significance (*P* < 0.05, *P* < 0.01, *P* < 0.001). These findings provide insight into the molecular mechanisms underlying PRV-induced neuropathology and potential therapeutic targets.

### Functional enrichment analysis of DEGs

**Figure 5:**
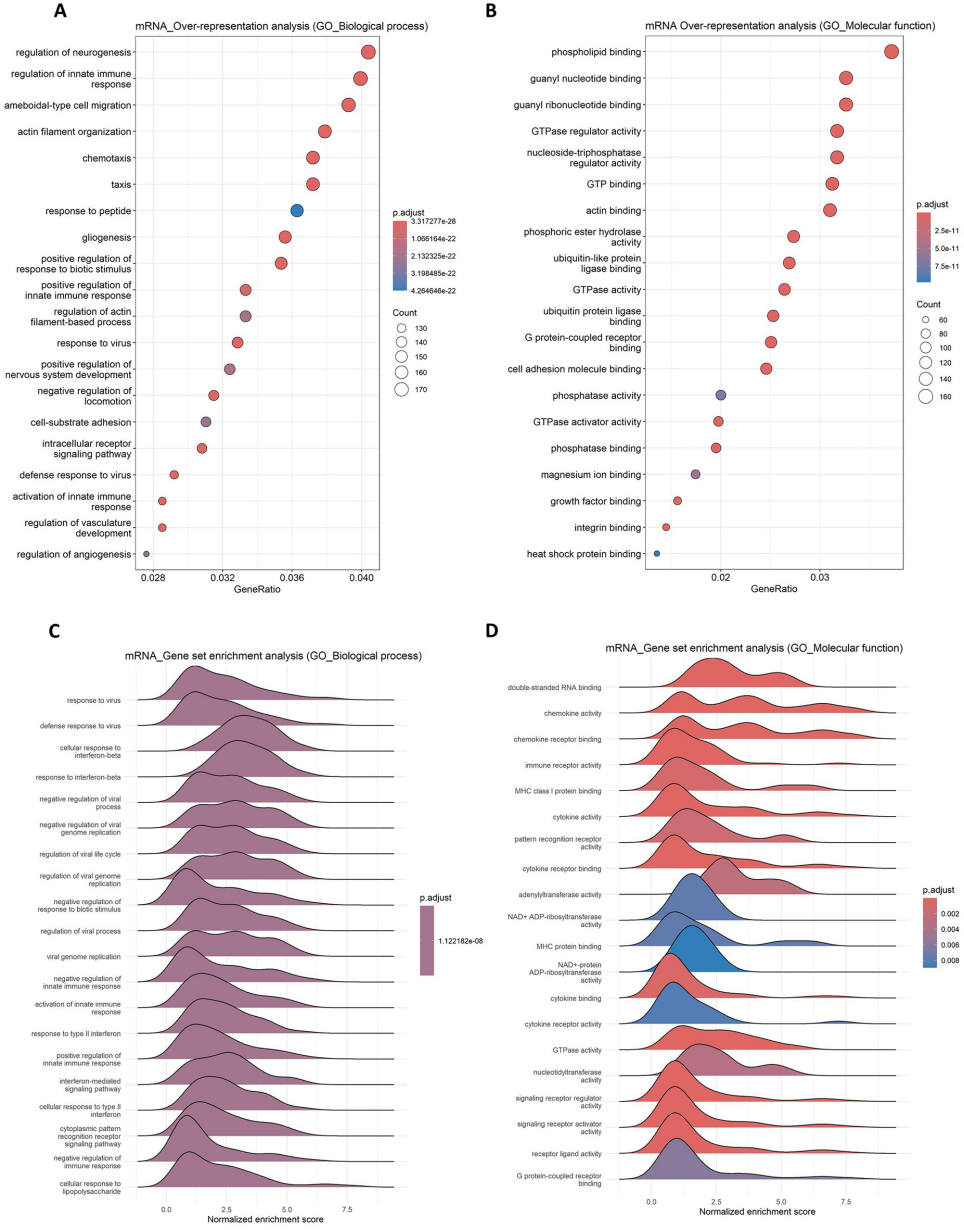
**Significantly enriched pathways altered in the brain in response to PRV infection**. Over-representation analysis (ORA) of Gene Ontology (GO) **A** biological processes and **B** molecular functions for DEGs in PRV-infected brains. Gene set enrichment analysis (GSEA) of GO **C** biological processes and **D** molecular functions in PRV-infected brain tissue.

Gene ontology (GO) pathway analysis revealed significant enrichment of biological processes and molecular functions related to immune activation, neurogenesis, and viral response in the brains of PRV-infected mice. Over-representation analysis (ORA) of differentially expressed genes (DEGs) identified key biological processes, including the innate immune response, neurogenesis, ameboid-type cell migration, chemotaxis, viral response, and angiogenesis/vascular regulation (Figure 5A). These findings indicate that PRV infection induces extensive immune activation and tissue remodeling in the brain. For molecular functions, ORA identified significant enrichment of phospholipid binding, guanine nucleotide binding, GTPase activity, actin binding, and phosphatase activity (Figure 5B), suggesting alterations in signaling pathways critical for immune responses and cellular communication during infection.

GSEA-based biological process analysis, visualized through ridge plots (Figure 5C), revealed prominent activation of gene sets involved in antiviral responses, including cellular responses to interferon-beta, interferon-mediated signaling, and general defense mechanisms against viral infections. These pathways exhibited the highest normalized enrichment scores (NES). Additionally, ridge plots highlighted significant enrichment of gene sets related to the negative regulation of viral genome replication, modulation of viral processes, activation of innate immune responses, and dual regulatory functions influencing innate immunity. In parallel, GSEA of molecular functions (Figure 5D) revealed enriched pathways, such as chemokine activity, cytokine receptor binding, pattern recognition receptor activity, and signaling receptor‒ligand interactions. Together, these findings highlight robust immune activation at levels of both biological process and molecular function during PRV infection.

### Diverse signaling alterations in innate and adaptive immunity

To examine the impact of PRV infection on key immune signaling pathways in the brain, we analyzed both adaptive and innate immune responses. KEGG pathway enrichment analysis and *PathView* visualized the four most affected pathways: antigen processing, toll-like receptor (TLR), retinoic acid-inducible gene I (RIG–I), and cytosolic DNA-sensing pathways.

#### Antigen processing and presentation pathways

The major histocompatibility complex class I (MHC–I) pathway is responsible for presenting intracellular viral antigens to CD8 + T–cells. The major histocompatibility complex class I (MHC–I) pathway was upregulated, as indicated by the increased expression of key genes, including proteasome activator complex 28 (PA28), transporter associated with antigen processing (TAP1/2), β2–microglobulin (β2m), and MHC–I (Additional file 7). In addition, CD8 expression was elevated, indicating activation of cytotoxic T–cells. In contrast, the major histocompatibility complex class II (MHC–II) pathway showed significant downregulation of cAMP response element-binding (CREB), nuclear transcription factor Y (NF–Y), CD4, and MHC–II genes, suggesting impaired antigen presentation to CD4 + helper T–cells. This suppression may represent a viral strategy to evade immune surveillance by reducing helper T–cell activation and cytokine production, as also observed in other herpesvirus infections [20, 21]. Furthermore, the killer-cell immunoglobulin-like receptor (KIR) gene was upregulated, indicating possible modulation of NK cell activity, potentially enhancing recognition and cytotoxic responses toward PRV-infected cells [22]. These findings highlight the complex interplay between PRV infection and host immune mechanisms, with both activation and suppression of specific pathways contributing to disease progression.

#### Toll-like receptor signaling pathway

**Figure 6:**
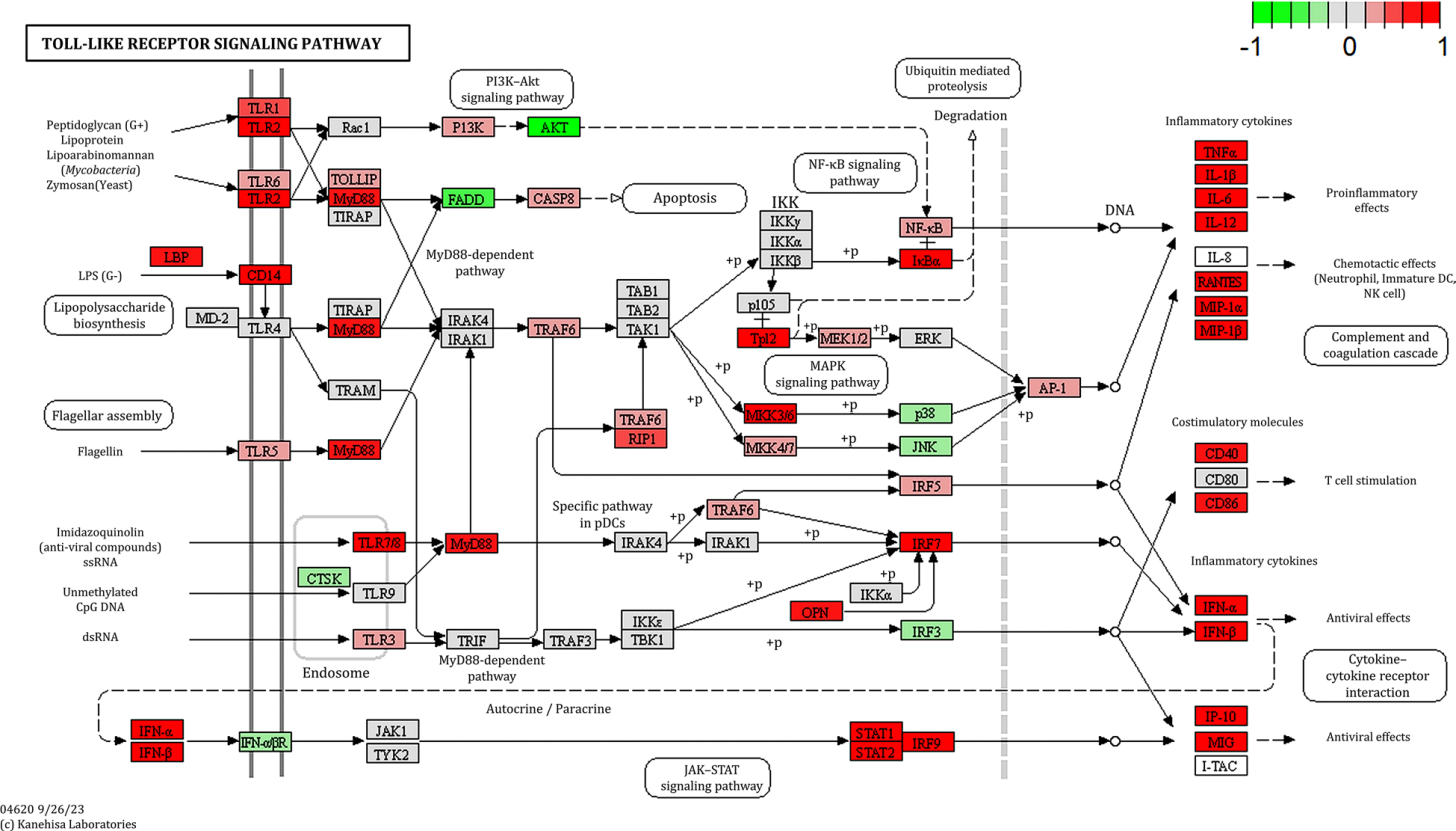
**Toll-like receptor (TLR) signaling pathway map overlaid with RNA-seq data.** The pathway diagram highlights differentially expressed genes (DEGs) involved in innate immune signaling in response to Pseudorabies virus (PRV) infection. Color intensity represents the log2 fold change in gene expression: red indicates upregulation and green indicates downregulation in infected brain tissue compared with controls.

Significant upregulation of multiple TLR genes, including TLR1, TLR2, TLR3, TLR6, and TLR7/8, suggests the strong host’s ability to recognize a broad spectrum of pathogen-associated molecular patterns (PAMPs), including envelope proteins, double-stranded DNA intermediates, and other virally derived components (Figure 6). Notably, TLR2, which is known to recognize viral glycoproteins, exhibited pronounced expression, consistent with findings in human cytomegalovirus (HCMV) [23]. This activation may trigger downstream MyD88-dependent signaling pathway, leading to the activation of transcription factors, such as nuclear factor kappa B (NF–κB), activator protein 1 (AP–1), and interferon regulatory factors (IRFs). There was robust upregulation of proinflammatory cytokine genes (TNF–α, IL–1β, and IL–6) and chemokine genes (RANTES, MIP–1α, and MIP–1β), reflecting a strong inflammatory response that recruits immune cells to the site of infection. Concurrently, increased interferon production (IFN–α and IFN–β) highlighted a potent antiviral state.

#### RIG–I-like receptor signaling pathway

The RIG–I-like receptors (RLRs), a family of pattern recognition receptors (PRRs), play a critical role in recognizing viral RNA and initiating innate immune responses. Despite PRV being a DNA virus, the upregulation of retinoic acid-inducible gene I (RIG–I) and melanoma differentiation-associated protein 5 (MDA5) gene expression was observed (Additional file 8). This activation promoted type I interferon production (IFN–α, IFN–β) through the activation of and interferon regulatory factor 7 (IRF7) and NF–κB. Additionally, proinflammatory cytokine genes, such as TNF–α and IL–12, were significantly upregulated, contributing to immune cell recruitment and the activation of adaptive immunity.

#### Cytosolic DNA-sensing pathway

As a DNA virus, PRV infection in brain tissue leads to a highly activated cytosolic DNA-sensing pathway (Additional file 9). Transcriptomic profiling revealed a significant upregulation of cyclic GMP–AMP synthase (cGAS), indicating the detection of viral DNA in the cytoplasm. Interestingly, the expression of STING (Stimulator of Interferon Genes), the adaptor downstream of cGAS, was slightly downregulated at the mRNA level. Despite this, we observed a robust increase in type I interferon production (IFN–α and IFN–β) and proinflammatory mediators (IL-6, CCL4, CCL5, and CXCL10). In addition, transcriptomic profiling revealed significant upregulation of several key mediators of PANoptosis, including Z-DNA-binding protein 1 (ZBP1), receptor-interacting serine/threonine-protein kinase 1 (RIPK1), mixed lineage kinase domain-like protein (MLKL), gasdermin-d (GSDMD), and IL-1β, indicating that PRV infection triggers a coordinated PANoptotic cell death program. This finding is consistent with evidence that ZBP1 serves as a central innate immune sensor capable of assembling PANoptosome complexes and driving inflammatory cell death during viral infection [24].

### Co-expression analysis and target prediction

**Figure 7:**
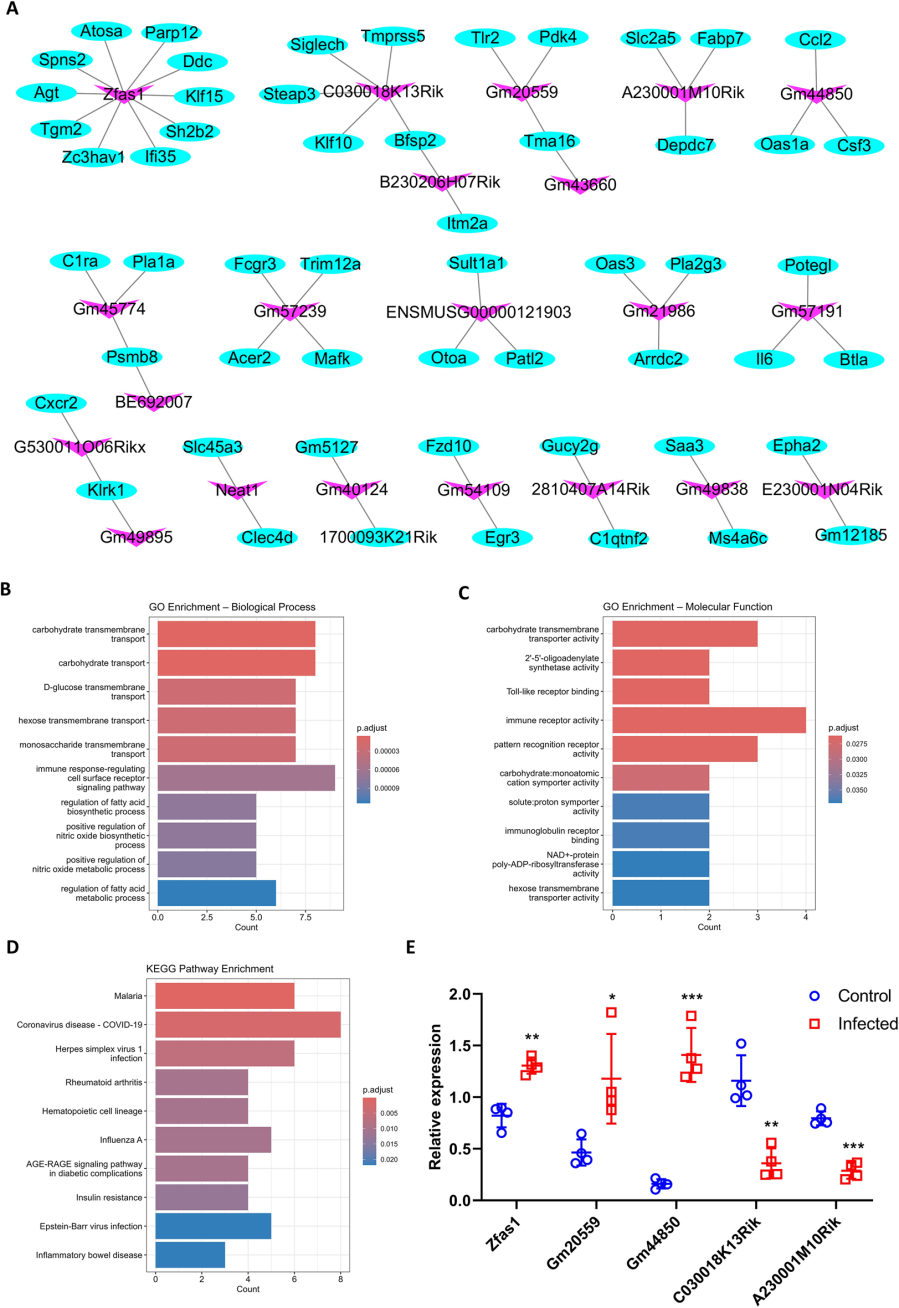
**Co-expression network of representative lncRNAs and their partial target mRNAs**. **A** Co-expression network showing representative lncRNAs (pink nodes), and their partially predicted target mRNAs (blue nodes). **B** Top 10 enriched GO biological process terms for target mRNAs. **C** Top 10 enriched GO molecular function terms for target mRNAs. **D** Top 10 enriched KEGG pathways for target mRNAs. **E** Validation of the expression levels for key lncRNA nodes (Zfas1, Gm20559, Gm44850, C0300018K13Rik, and A230001M10Rik) via RT‒qPCR. Relative lncRNA expression levels are shown for control (blue dots) and infected (red dots) samples. β–actin was used as the internal control gene. The results are presented as the mean ± SD; *P < 0.05, **P < 0.01, ***P < 0.001, and ****P < 0.0001, compared with the control group (multiple *t* tests, *n* = 4).

A co-expression network was constructed to explore the interactions between DElncRNAs and their potential target DEmRNAs during PRV infection. This analysis enabled the identification of key lncRNAs by inferring functional links with mRNAs on the basis of their correlated expression patterns across samples [25–27]. The network revealed representative lncRNAs (pink nodes) and their partially predicted target mRNAs (blue nodes), forming a complex regulatory structure (Figure 7A, Additional file 10). A total of 43 lncRNAs and 77 mRNAs were identified to form 81 significant co-expression pairs, defined by an absolute Pearson correlation coefficient (|PCC|) > 0.99 and *P* < 0.05. Notable lncRNA nodes, such as Zfas1, Gm20559, Neat1, C0300018K13Rik, and A230001M10Rik, were identified as central hubs, connecting to multiple target mRNAs, highlighting these transcripts as key lncRNAs in PRV-induced encephalitis. For example, lncRNA Zfas1 was found to be correlated with 10 mRNAs (Atosa, Agt, Tgm2, Zc3hav1, Ifi35, Sh2b2, Klf15, Ddc, Parp12, and Spns2). Furthermore, functional enrichment analysis of these target mRNAs revealed significant enrichment in immune response regulation, carbohydrate transmembrane transport, and the nitric oxide biosynthetic process, based on GO biological process analysis (Figure 7B). Molecular function analysis further indicated predominant enrichment in immune receptor activity, carbohydrate transmembrane transporter activity, and Toll-like receptor binding (Figure 7C). KEGG pathway analysis also showed enrichment in infectious disease pathways, particularly those related to COVID-19, herpes simplex virus 1 infection, and other viral response mechanisms, suggesting that these genes may be involved in general antiviral responses (Figure 7D). The expression levels of several lncRNA nodes were further validated using RT‒qPCR (Figure 7E). Significant alterations in Zfas1, Gm20559, Gm44850, C0300018K13Rik, and A230001M10Rik were observed in the PRV-infected brain compared with controls, consistent with the RNA–seq data. These results suggest the involvement of lncRNAs in the host response to PRV infection, and point to potential central roles in the co-expression network.

### PRV infection elicits dose-, time-, and cell type–dependent lncRNA responses

To investigate the regulatory dynamics of lncRNAs in response to PRV infection at the cellular level, BV2 microglial cells and N2a neuron-like cells were infected at MOI of 0.5 and 2. Total RNA was collected at 6 and 12 hours post-infection (hpi) for RT–qPCR analysis of proinflammatory cytokines and selected lncRNAs.

**Figure 8:**
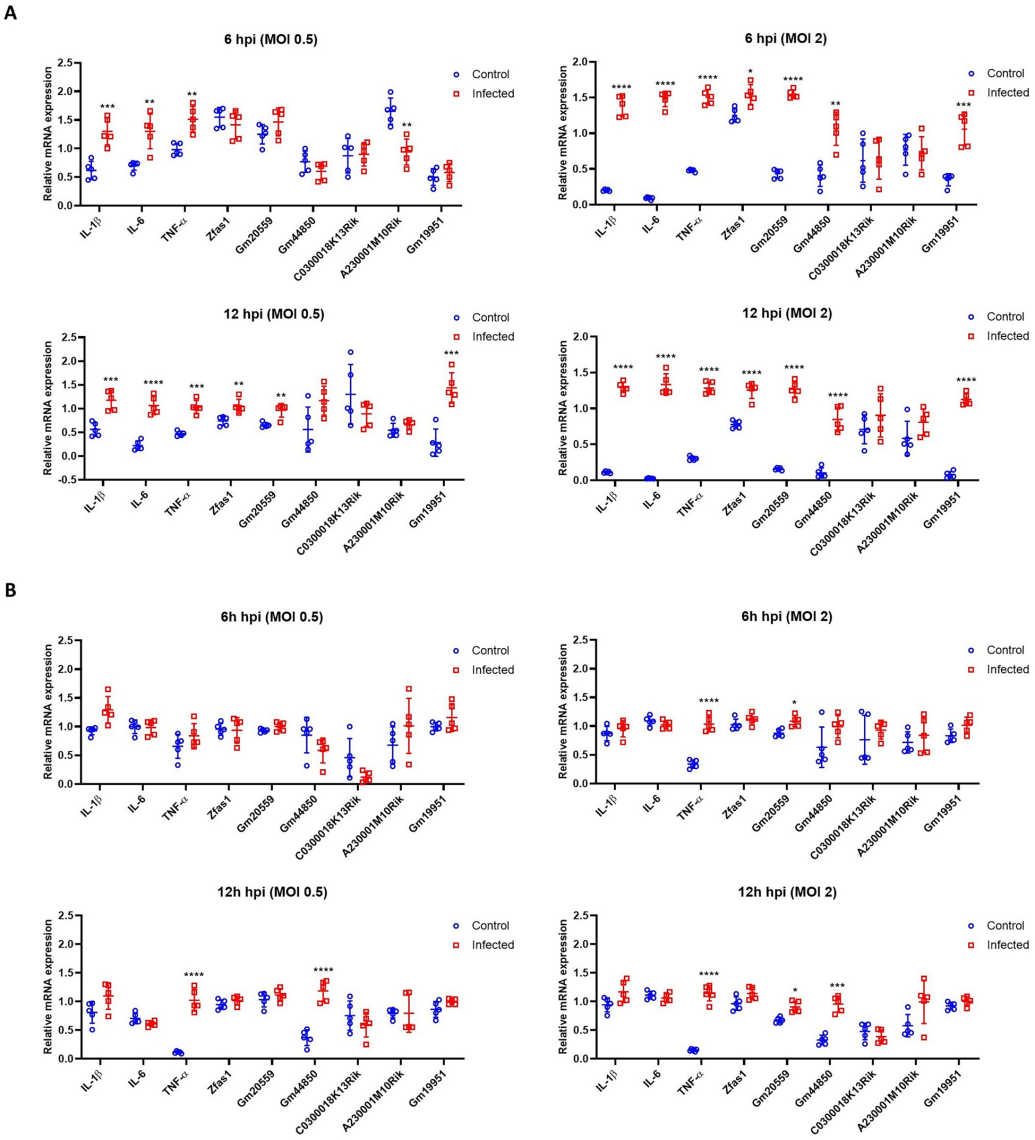
**PRV induces the expression of lncRNAs in mouse microglial and neuronal cells**. BV2 microglial **A** and N2a neuron-like **B** cells were infected with PRV at a multiplicity of infection (MOI) of 0.5 and MOI of 2, and cells were harvested at 6- and 12-h post-infection (hpi). The expression levels of proinflammatory cytokines (IL–1β, IL–6, TNF–α) and selected lncRNAs (Zfas1, Gm20559, Gm44850, C0300018K13Rik, A230001M10Rik, and Gm19951) were analyzed by RT‒qPCR. β–actin was used as the internal control gene, and five biological replicates were analyzed. Data displayed as mean ± SD; *P < 0.05, **P < 0.01, ***P < 0.001, and ****P < 0.0001, compared with the control group (multiple *t* tests, *n* = 5).

In BV2 microglial cells, the primary immune cells of the CNS, PRV infection induced significant inflammatory and lncRNA responses in a dose- and time-dependent manner (Figure 8A). At MOI of 0.5, the expression of proinflammatory cytokines IL–1β, IL–6, and TNF–α was significantly upregulated at both 6 and 12 hpi. In parallel, lncRNAs Zfas1, Gm20559, and Gm19951 were markedly elevated by 12 hpi. At MOI of 2, these responses were further amplified, with higher expression levels of cytokines and lncRNAs (Zfas1, Gm20559, Gm44850, and Gm19951) at both 6 and 12 hpi, highlighting a dose-dependent enhancement of microglial inflammatory signaling and lncRNA activation.

By contrast, N2a neuron-like cells exhibited a more limited and delayed transcriptional response (Figure 8B). At MOI of 0.5, no significant changes were observed in either cytokine or lncRNA expression at 6 hpi, while TNF–α and Gm44850 showed significantly increased expression by 12 hpi, indicating a delayed and selective activation. At MOI of 2, TNF–α and Gm20559 were upregulated at 6 hpi, and by 12 hpi, a more pronounced induction of TNF–α, Gm20559, and Gm44850 was observed. These findings suggest that a higher viral load accelerates and amplifies the neuronal transcriptional response, involving both inflammatory cytokines and regulatory lncRNAs. Collectively, these results demonstrate that PRV infection elicits dynamic lncRNA expression changes dependent on dose, time, and cell type.

### Regulation of microglia-mediated inflammation by host lncRNA ZFAS1 upon PRV infection

**Figure 9:**
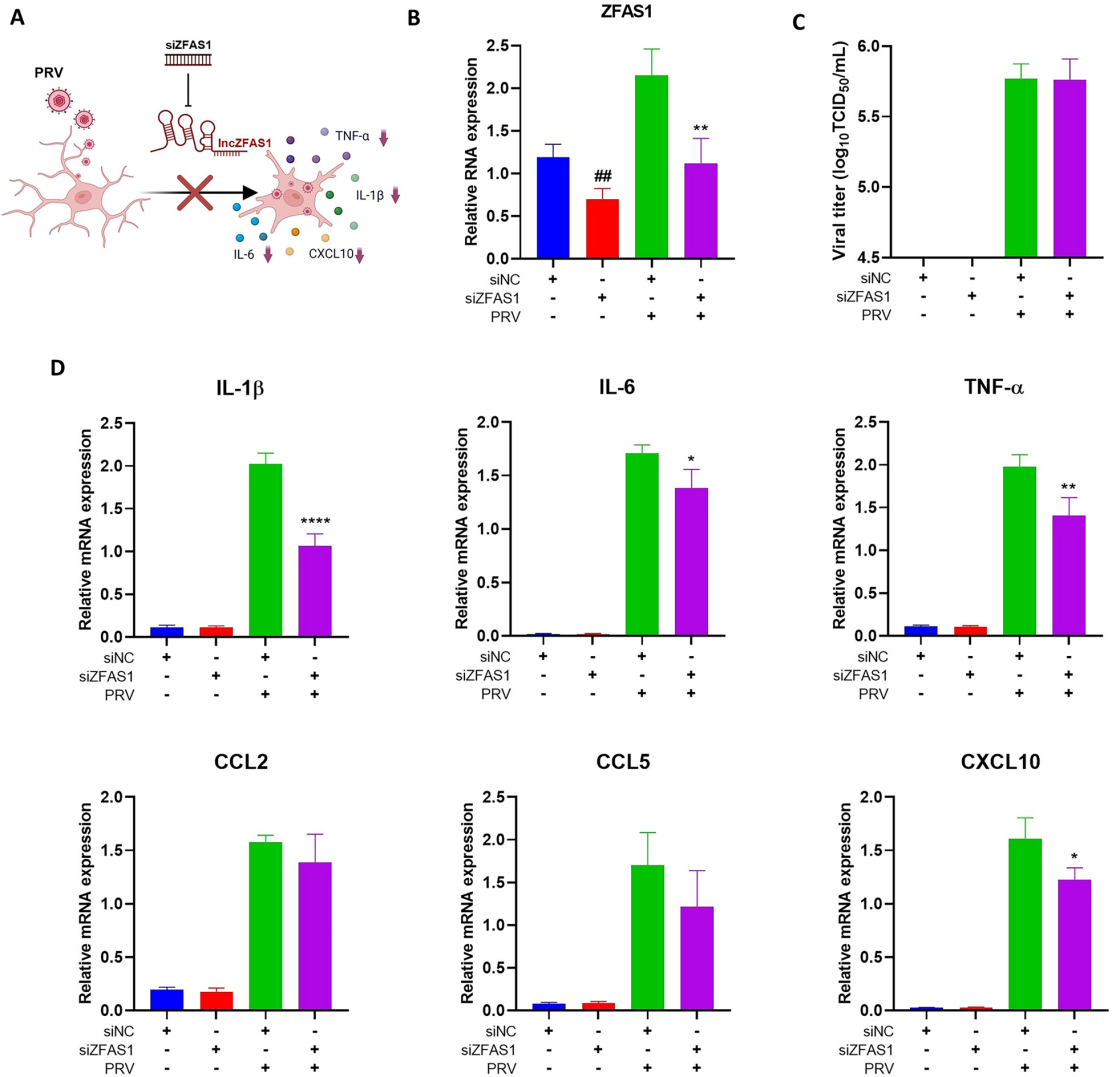
**ZFAS1 regulates microglial inflammatory responses upon PRV infection**. BV2 microglial cells were transfected with control siRNA (siNC) or ZFAS1-specific siRNA (siZFAS1) and subsequently infected with or without PRV. Gene expression was analyzed by RT‒qPCR and normalized to β–actin, while viral titers were determined by TCID_50_ assay. **A** Diagram showing that PRV infection upregulates lncZFAS1, enhancing inflammatory cytokine production in microglia, while siRNA-mediated knockdown (siZFAS1) reduces this response. Created with BioRender. **B** Relative mRNA expression of ZFAS1 measured by RT‒qPCR (unpaired *t* test, *n* = 4). **C** TCID_50_ assay detection of virus titer (unpaired *t* test, *n* = 4). **D** Relative mRNA expression levels of proinflammatory cytokines IL–1β, IL–6, and TNF–α, and chemokines CCL2, CCL5, and CXCL10 in BV2 microglial cells (unpaired *t* test, *n* = 4). Data represent mean ± SD, *P < 0.05, **P < 0.01, ****P < 0.0001 versus PRV + siNC group; ^##^*P* < 0.01 versus siNC uninfected group.

Through differential expression analysis and co-expression network modeling of lncRNA-mRNA interactions, we identified ZFAS1 as one of the most significantly dysregulated lncRNAs, with the highest number of co-expressed edges, suggesting a potential regulatory hub in PRV-induced neuroinflammation. To explore its function, we performed siRNA-mediated knockdown of ZFAS1 in BV2 microglial cells. The results showed that silencing ZFAS1 significantly reduced its expression in both PRV-infected and uninfected cells (Figure 9b). ZFAS1 knockdown did not significantly affect PRV infection, as indicated by comparable TCID_50_ viral titers (Figure 9C). Analysis of key inflammatory mediators revealed that PRV infection strongly upregulated the expression of proinflammatory cytokines IL–1β, IL–6, and TNF–α, as well as chemokines CCL2, CCL5, and CXCL10, compared with uninfected controls (Figure 9D). Notably, ZFAS1 silencing significantly attenuated the PRV-induced expression of these cytokines and CXCL10, suggesting a pivotal role for ZFAS1 in amplifying microglial inflammatory responses during PRV infection (Figure 9D). Figure 9A provides an overview of the ZFAS1-mediated inflammatory pathway confirmed by our findings.

## Discussion

Pseudorabies virus (PRV) poses a significant threat to the swine industry, and raises concerns about zoonotic transmission [7]. Despite extensive research efforts, effective antiviral treatments remain unavailable, and owing to the emergence of resistant viral strains, vaccines are becoming increasingly ineffective [28, 29]. Increasing reports of PRV infections in human, including cases of severe PRE associated with hSD–1/2019 variant strains, underscore the urgent need for deeper insights into PRV pathogenesis [3]. Previous transcriptomic studies were limited by their reliance on gene probes that predominantly capture highly abundant mRNAs, while overlooking low expressed mRNAs and lncRNAs, which are known to play critical roles in host‒pathogen interactions [9, 10]. Advances in high-throughput RNA sequencing and bioinformatic analysis have enabled more comprehensive analyses of PRV‒host interactions in several in vitro models [30, 31]. A recently published study identified several lncRNAs; however, differences in reference gene annotations and the lack of functional evidence pose significant challenges for cross-study comparison and interpretation [32]. The present study provides a comprehensive transcriptomic analysis of PRV infection in a mouse model, along with experimental validation of selected lncRNAs at the cellular level. This approach offers a deeper understanding of host‒pathogen interactions during PRE, and identifies novel lncRNA therapeutic targets to modulate immune responses.

Since the pathogenicity of PRV varies depending on the infection route, we used the intranasal route to mimic the natural oronasal route of infection in mammals [33]. Consistent with previous studies, survival analysis revealed that infected mice began dying on 4 dpi, with complete mortality by 5 dpi [34]. Clinical observations showed significant weight loss and neurological symptoms starting at 3 dpi, suggesting that by this time point, the virus had invaded the brain. Consequently, we designated 3 dpi as the optimal time for brain sample collection. Supporting the effectiveness of this model, both TCID_50_ quantification and PRV immunohistochemistry (IHC) confirmed robust viral replication and widespread neuronal infection in the brain. This finding was further corroborated by the robust activation of proinflammatory cytokine genes, such as IL–1β, IL–6, and TNF–α, and chemokines including CCL2, CCL5, and CXCL10, as well as pronounced Iba1 + myeloid cell activation in affected brain regions. These findings are consistent with those of other neurotropic viral infections, such as HSV and JEV, which show strong neuroinvasion and transneuronal spread following intranasal infection, but with a shorter survival time [35, 36]. Notably, previous studies in pigs reported extensive PRV replication in the olfactory bulbs, accompanied by robust cytokine mRNA upregulation [37]. In addition, a human case of PRV encephalitis revealed marked immune cell infiltration (including neutrophils, lymphocytes, and monocytes) into the cerebrospinal fluid [5], strengthening the cross-species relevance of our findings in the mouse model to PRV neuropathogenesis.

Although the use of bulk brain tissue for transcriptomic analysis may reduce the resolution to identify effector pathways specific to individual brain regions, we reasoned that PRV infection would be widespread, allowing prominent pathways to still be clearly identified. Notably, the diversity and abundance of detected RNA species underscore the complexity of host responses at the genomic level during neurotropic viral infections. Furthermore, heatmap clustering and PCA analyses revealed distinct clusters for both mRNA and lncRNA profiles between infected and control samples, strongly suggesting a clear regulatory role for lncRNAs during PRV-induced encephalitis. Similar observations of significant transcriptional reprogramming have been reported in studies of other neurotropic viruses, such as the West Nile virus (WNV) and Chikungunya virus (CHIKV), which also demonstrate clear clustering of infected and control brains [38]. However, differences in the magnitude of variance explained by PC1 and PC2 between mRNA and lncRNA ((91 cf. 68) %) profiles in our analysis suggest that coding genes exhibit a more uniform and robust response than noncoding RNAs, possibly due to functional differences and the varied regulatory roles of lncRNAs [14]. These findings were supported by the high quality of RNA–seq data, as indicated by excellent RNA integrity and sequencing metrics, which ensured reliable detection of both coding and noncoding RNAs. Moreover, the mouse model benefits from comprehensive and well-annotated genomes for both protein-coding and noncoding RNAs, facilitating confident identification and interpretation of transcriptomic changes [39, 40]. While the mouse model provides essential insights into PRV-induced encephalitis, it may not fully recapitulate the host-specific responses observed in other species. Future cross-species RNA–seq studies—including in pig and human-derived tissues—will be essential for advancing translational research on PRV neuropathogenesis.

The DEG analysis identified 683 DEmRNAs and 179 DElncRNAs, highlighting a profound host genomic response to viral infection. Notably, the enrichment of both DEmRNAs and DElncRNAs on chromosome 7 suggests that PRV infection disrupts chromosomal hotspots potentially regulated by genes critical for antiviral defense or neuronal function. Immune-related genes, such as Cxcl2, Cxcl9, Cxcr2, Il1rn, Trem1, and Oas3, were significantly upregulated, indicating a robust inflammatory response. The downregulation of olfactory receptor-related genes, such as Or4d1 and Or14j4, suggests potential PRV entry through the olfactory nerve pathway and/or significant damage to the olfactory epithelium and associated neural structures. Current evidence demonstrates that neurotropic herpesviruses utilize multiple concurrent CNS entry routes, with viral infections causing substantial downregulation of olfactory receptor genes as both a consequence of viral entry and a marker of olfactory epithelial damage [41–45]. While the olfactory pathway serves as one documented route of viral neuroinvasion, the trigeminal nerve pathway appears to be predominant for many herpesvirus CNS infections following intranasal exposure [41, 44]. The relative importance of these pathways varies by virus type, host factors, and infection conditions, emphasizing the need for comprehensive assessment of CNS entry mechanisms rather than attribution to a single pathway. Additionally, the downregulation of Rpe65 (retinal pigment epithelium-specific 65 kDa) may contribute to the visual impairment symptoms commonly observed in PRV patients. Fortunately, the FDA-approved gene therapy Luxturna, which targets Rpe65, could be considered a potential therapeutic option for post-PRV visual dysfunction [46]. Interestingly, Gkn3 (gastrokine 3), which has previously been reported to exhibit elevated expression and function as a host receptor facilitating JEV entry into neurons in the mouse brain, was significantly downregulated during PRV infection, highlighting distinct virus-specific host interactions [47]. Unlike protein-coding genes, lncRNAs are typically not conserved in their sequence motifs and secondary structures, which makes it more challenging to predict their functions [48]. Several DElncRNAs, including 9330175E14Rik, may be involved in pro-inflammatory pathways, while downregulated lncRNAs, such as C030029H02Rik, could play a role in the regulation of oligodendrocyte function [49–51]. RT–qPCR further validated the notable changes identified in DEmRNAs and DElncRNAs, highlighting their potential as therapeutic targets or biomarkers for PRV-induced encephalitis.

The key pathways in the PRE, involving innate immunity, chemotaxis, angiogenesis, and neurogenesis, are also well-known in other neurotropic viral infections, such as rabies virus (RABV) and WNV [38, 52]. Interestingly, KEGG pathway analysis revealed simultaneous activation and suppression of immune pathways, including antigen processing and presentation, Toll-like receptor (TLR) signaling, RIG–I-like receptor signaling, and cytosolic DNA-sensing pathways. The strong activation of multiple TLRs observed at 3 dpi suggests a severe encephalitic response in the mouse brain, which may contribute to the rapid mortality observed in PRV-infected mice. Despite PRV being a DNA virus, PRV infection induces the upregulation RIG-I and MDA5 gene expression, potentially due to the recognition of viral replication intermediates, as part of a typical innate immune response observed in herpesvirus infections [53]. In addition, genes related to MHC-I antigen processing were upregulated, whereas those associated with MHC-II pathways were downregulated, potentially representing a viral strategy to evade helper *T*–cell-mediated immunity. This modulation has been observed in other members of the herpesvirus family, such as HSV-1 and human cytomegalovirus (HCMV), as well as in human immunodeficiency virus (HIV), where selective suppression of MHC–II pathways aids immune evasion [20, 21, 54]. Extending this immune evasion strategy, PRV utilizes its UL13 protein to inhibit STING-mediated antiviral signaling by promoting STING ubiquitination and degradation [55]. Moreover, our GSEA analysis highlights the prominence of interferon-mediated pathways, suggesting a critical role for type I and type II interferons in controlling viral replication within the CNS. Notably, the enrichment of processes related to negative regulation of viral replication and dual regulatory mechanisms of innate immunity highlight the active effort of the CNS to mitigate the pathogenic effects of the virus. Future studies using single-cell or spatial transcriptomics could further elucidate specific cell types and brain regions involved in driving these immune responses, and examine the consequences of prolonged immune activation on neuroinflammation and neuronal integrity.

LncRNAs play essential roles in various biological processes and diseases, offering potential therapeutic applications [14]. Although the involvement of lncRNAs in infectious diseases has been increasingly recognized, few studies have specifically explored their roles in PRV pathogenesis [18, 56]. Our study represents the comprehensive analysis of lncRNA expression changes in PRE. Unique clustering patterns and 179 DElncRNAs were observed in the lncRNA transcriptomic profiles between infected and control samples, suggesting the crucial and diverse roles of lncRNAs during PRV pathogenesis. Further support for their functional significance was provided by co-expression network analysis, which identified key lncRNAs, such as Zfas1, Neat1, C030018K13Rik, Gm20559, Gm44850, and A230001M10Rik, as central regulatory hubs coordinating the expression of immune system-related genes and neurotransmitter synthesis. Notably, some of these lncRNAs, such as Neat1, have previously been associated with cytokine storms in both moderate and severe forms of COVID–19 infection [57]. Gm20559 also plays a crucial role in regulating the inflammatory environment of microglia during flaviviral infection [17]. Zfas1 has been shown to positively regulate the antiviral innate immune response during infection with vesicular stomatitis virus (VSV) and herpes simplex virus type 1 (HSV–1) [58]. The downregulation of lncRNA C030018K13Rik aligns with its reduced expression after traumatic brain injury, indicating a potential common role in neural response to damage [59]. Also, several previously unreported lncRNAs form critical co‒expression networks with functional mRNAs. The downregulated lncRNA A230001M10Rik is strongly correlated with Fabp7, Slc2a5, and Depdc7, which might suggest potential roles in neural homeostasis, glial metabolism, and cellular proliferation during infection [60–62]. The upregulated lncRNA Gm44850 is associated with Ccl2, Oas1a, and Csf3, indicating its involvement in neuroinflammatory responses and antiviral defense mechanisms through possible regulation of immune cell recruitment and interferon-stimulated activity [63, 64]. RT‒qPCR results further confirmed the significant changes in these lncRNAs in mouse brain. In addition, functional enrichment analysis was performed on their predicted target mRNAs and showed significant enrichment in immune regulation, viral response pathways, and neuroinflammatory processes, reinforcing their potential as critical regulators of key host defense mechanisms during PRV infection.

Furthermore, in vitro experiments using mouse microglial and neuron-like cells revealed the cell type-specific nature of lncRNA regulation during PRV infection, with microglia exhibiting a more rapid and diverse lncRNA response compared with neurons among the subset of lncRNAs examined. Notably, ZFAS1 emerged as a significantly dysregulated lncRNA in both PRV-infected mouse models and BV2 microglial cells, displaying the highest number of co-expression edges among the lncRNAs analyzed. This suggests that ZFAS1 may act as a central regulatory hub in PRV-induced encephalitis. Although previous studies have implicated ZFAS1 in mediating apoptosis in myocardial infarction models and promoting metastasis in hepatocellular carcinoma, its functional role during viral encephalitis remains unclear [65, 66]. Our findings demonstrate that ZFAS1 contributes to microglia-mediated inflammatory responses during PRV infection, as its knockdown markedly reduced the expression of key proinflammatory cytokines and chemokines without altering viral titers. This indicates that ZFAS1 primarily modulates host inflammatory pathways rather than directly influencing viral propagation. Furthermore, ZFAS1 has been previously associated with neuroinflammatory conditions such as temporal lobe epilepsy and neuropathic pain, suggesting that its regulatory functions may extend to broader CNS pathologies [67, 68]. Considering that herpesviruses have been linked to neurological disorders including mesial temporal lobe epilepsy and multiple sclerosis [69, 70], ZFAS1 may represent a potential therapeutic target for modulating neuroinflammation in virus-associated neurological diseases.

In conclusion, this study presents a comprehensive transcriptomic analysis of pseudorabies encephalitis in a mouse model, offering critical insights into PRV pathogenesis and host‒pathogen interactions. By integrating bioinformatic approaches and experimental validation, we identified several central regulatory lncRNAs which may serve as key modulators of glial metabolism and immune responses. Notably, ZFAS1 emerged as a significantly interactive lncRNA, demonstrating regulation of microglia-mediated inflammation and representing a promising therapeutic target for modulating neuroinflammation in virus-associated neurological diseases. These findings highlight the crucial role of lncRNAs in shaping host responses during PRE, and lay the foundation for developing novel therapeutic strategies targeting PRV infection and its neurological sequelae.

## Supplementary Information

**Additional file 1.** **Primer list.**

**Additional file 2.** **Quality control of RNA and sequencing.**

**Additional file 3.** **Clinical scores of mice intranasally infected with PRV.**

**Additional file 4 Distribution of gene types in the transcriptomic data**. (A) Distribution of original gene types detected in RNA–seq data from mouse brain samples. (B) Distribution after similar gene types were merged into broader categories. (C) Distribution of mRNAs across chromosomes. (D) Distribution of lncRNAs across chromosomes.

**Additional file 5**. **Top 20 upregulated and downregulated DEmRNAs.**

**Additional file 6.** **Top 20 upregulated and downregulated DElncRNAs.**

**Additional file 7**. **Antigen processing and presentation pathways in PRE.**

**Additional file 8.** **RIG−I-like receptor signaling pathway in PRE**.

**Additional file 9.** **Cytosolic DNA-sensing pathway in PRE.**

**Additional file 10.** **Detailed top correlations between lncRNAs and mRNAs.**

## Data Availability

The transcriptome data can be accessed from the SRA database under the accession number PRJNA1254890 [71].

## Abbreviations

CNS: central nervous system
DEGs: differentially expressed genes
DElncRNAs: differentially expressed long noncoding RNAs
DEmRNAs: differentially expressed mRNAs
GO: Gene Ontology
KEGG: Kyoto Encyclopedia of Genes and Genomes
lncRNA: long noncoding RNA
lncRNAs: long noncoding RNAs
mRNAs: messenger RNAs
ORA: over-representation analysis
PCA: principal component analysis
PRE: pseudorabies encephalitis
PRV: pseudorabies virus
RNA–seq: RNA sequencing
RT‒qPCR: reverse transcription quantitative polymerase chain reaction
