# Supplementary material for: Transcriptomic landscape of pseudorabies virus-induced encephalitis reveals key lncRNAs involved in host–neurotropic virus interactions
Source: Vet Res. 2025 Nov 10;56:216. doi: 10.1186/s13567-025-01650-5 (PMC12604289; doi:10.1186/s13567-025-01650-5)
Supplement: Supplementary file 1 — Additional file 1. Primer list. [file 13567_2025_1650_MOESM1_ESM.docx]

**Additional file 1: Primer list.**

| **Gene** | **Gene_type** | **Forward** | **Reverse** |
| --- | --- | --- | --- |
| TNF–α | mRNA | AGGGTCTGGGCCATAGAACT | CCACCACGCTCTTCTGTCTAC |
| IL–1β | mRNA | GGTCAAAGGTTTGGAAGCAG | TGTGAAATGCCACCTTTTGA |
| IL–6 | mRNA | ACCAGAGGAAATTTTCAATAGGC | TGATGCACTTGCAGAAAACA |
| CXCL10 | mRNA | ATCATCCCTGCGAGCCTATCC | TGTCCATCCATCGCAGCAC |
| CCL2 | mRNA | AGCAGCAGGTGTCCCAAAGA | GTGCTGAAGACCTTAGGGCAGA |
| CCL5 | mRNA | CCCTCACCATCATCCTCACT | CTTCTTCTCTGGGTTGGCAC |
| Slc10a6 | mRNA | GCTTCGGTGGTATGATGCTT | CCACAGGCTTTTCTGGTGAT |
| Cxcl2 | mRNA | ATGCCTGAAGACCCTGCCAAG | GGTCAGTTAGCCTTGCCTTTG |
| Rpe65 | mRNA | TCTCTGTTGCTGGAAAGGGT | TTGTATGGGGCAGTGTGACT |
| Saa3 | mRNA | CGCAGCACGAGCAGGAT | TGGCTGTCAACTCCCAGG |
| Gkn3 | mRNA | AGACAACATGAGACGCCTTATTG | CTGTCGCTAGTGTTCGTCAGC |
| Or4d1 | mRNA | TCTACACGCTCAGGAACCAG | AAGTCCAGCGAACTCTGTCA |
| β-actin | mRNA | TGGAATCCTGTGGCATCCATGAAAC | TAAAACGCAGCTCAGTAACAGTCCG |
| Gm35287 | lncRNA | ACAAGAAACGGGGAGCTCAG | GAGCCGAGCCTTCCATAGTC |
| 9330175E14Rik | lncRNA | GTGGATTCAAAGTGCCAGCCC | CACTGGACCCTGGACCCAAC |
| Gm19951 | lncRNA | GGCTCGAGGTACAAACCCTT | TGTGCAAACGGCGAGATGAT |
| Gm12324 | lncRNA | TCCTCGGTTACCCTGGTTCT | TGGATTGGCAGCAGCATACA |
| C030029H02Rik | lncRNA | TCAGACGTAACCAACGCACA | GGGTCCACCAGTTCCCAAAG |
| Zfas1 | lncRNA | AGCGTTTGCTTTGTTCCC | CTCCCTCGATGCCCTTCT |
| C030018K13Rik | lncRNA | TTCTGGCTGGTGTCCTAA | TGATCCTCACGCATCTCA |
| Gm20559 | lncRNA | ACCCTTGTAGGCCAAAGTAAG | CTATGGCTCTTGAGGAGTTTGT |
| A230001M10Rik | lncRNA | ACCCAGCTTGAATGGGATTTG | TTCCTGCATGCTGCCATGTT |
| Gm44850 | lncRNA | TGGAACTTCCTGTGAATGGCT | TGCAGTTTCCCTGGTGAGTTT |
