# Supplementary material for: Transcriptomic landscape of pseudorabies virus-induced encephalitis reveals key lncRNAs involved in host–neurotropic virus interactions
Source: Vet Res. 2025 Nov 10;56:216. doi: 10.1186/s13567-025-01650-5 (PMC12604289; doi:10.1186/s13567-025-01650-5)
Supplement: Supplementary file 2 — Additional file 2. Quality control of RNA and sequencing. [file 13567_2025_1650_MOESM2_ESM.docx]

**Additional file 2: Quality control of RNA and sequencing.**

| **Sample Name** | **Conc.**  **(ng/uL)** | **260/280** | **RIN** | **Total Reads** | **Q20 (%)** | **Q30 (%)** |
| --- | --- | --- | --- | --- | --- | --- |
| Control 1 | 637 | 2.11 | 7.5 | 65,868,392 | 97.9 | 94.6 |
| Control 2 | 622 | 2.06 | 7.5 | 59,505,104 | 98.1 | 94.9 |
| Control 3 | 647 | 2.09 | 7.3 | 89,149,852 | 98.3 | 95.4 |
| Control 4 | 631 | 2.08 | 7.4 | 88,943,444 | 98.3 | 95.4 |
| Infected 1 | 642 | 2.1 | 7.3 | 65,395,410 | 98.1 | 94.9 |
| Infected 2 | 646 | 2.11 | 7.2 | 88,723,664 | 98.3 | 95.3 |
| Infected 3 | 638 | 2.1 | 7.3 | 60,631,556 | 98.1 | 94.9 |
| Infected 4 | 625 | 2.1 | 7.3 | 87,830,250 | 98.3 | 95.3 |
