# Supplementary material for: Transcriptomic landscape of pseudorabies virus-induced encephalitis reveals key lncRNAs involved in host–neurotropic virus interactions
Source: Vet Res. 2025 Nov 10;56:216. doi: 10.1186/s13567-025-01650-5 (PMC12604289; doi:10.1186/s13567-025-01650-5)
Supplement: Supplementary file 3 — Additional file 3. Clinical scores of mice intranasally infected with PRV. [file 13567_2025_1650_MOESM3_ESM.docx]

**Additional file 3. Clinical scores of mice intranasally infected with PRV.**

| **Clinical Scores of Mice Intranasally Infected with PRV** | | | | | | | |
| --- | --- | --- | --- | --- | --- | --- | --- |
| **Category** | **Criteria** | **Score** | **1 dpi** | **2 dpi** | **3 dpi** | **4 dpi** |  |
| **General Appearance** | Normal appearance | 0 | 5/5 |  |  |  |  |
|  | Ruffled fur or mild signs of discomfort | 1 |  | 5/5 |  |  |  |
|  | Moderate ruffled fur, hunched posture | 2 |  |  | 2/5 |  |  |
|  | Severe ruffled fur, hunched posture, itching, skin Bites | 3 |  |  | 3/5 | 3/3 |  |
| **Neurological Signs** | No signs of neurological deficits | 0 | 5/5 | 5/5 |  |  |  |
|  | Slight unsteady gait, tremors | 1 |  |  | 1/5 |  |  |
|  | Moderate ataxia, tremors, head tilt, circling | 2 |  |  | 3/5 |  |  |
|  | Severe ataxia, paralysis, seizures | 3 |  |  | 1/5 | 3/3 |  |
| **Behavior** | Normal activity and behavior | 0 | 5/5 |  |  |  |  |
|  | Slightly reduced activity | 1 |  | 5/5 |  |  |  |
|  | Markedly reduced activity, less responsive, itching | 2 |  |  | 4/5 | 2/3 |  |
|  | Unresponsive, does not react to stimuli | 3 |  |  | 1/5 | 1/3 |  |
