# Supplementary figures and images for: Transcriptomic landscape of pseudorabies virus-induced encephalitis reveals key lncRNAs involved in host–neurotropic virus interactions
Source: Vet Res. 2025 Nov 10;56:216. doi: 10.1186/s13567-025-01650-5 (PMC12604289; doi:10.1186/s13567-025-01650-5)

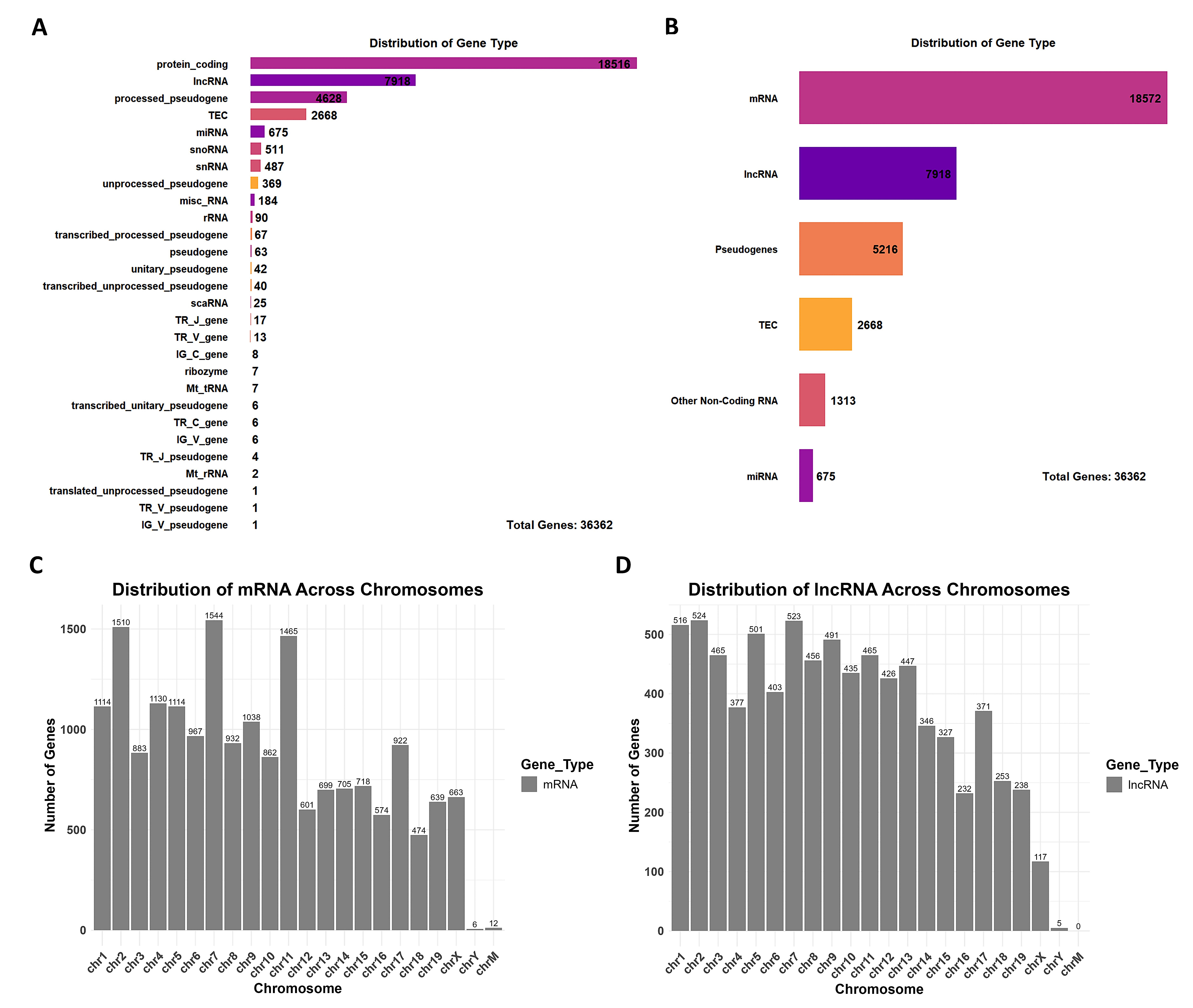

Supplement: Supplementary file 4 — Additional file 4 Distribution of gene types in the transcriptomic data. (A) Distribution of original gene types detected in RNA–seq data from mouse brain samples. (B) Distribution after similar gene types were merged into broader categories. (C) Distribution of mRNAs across chromosomes. (D) Distribution of lncRNAs across chromosomes. [file 13567_2025_1650_MOESM4_ESM.jpg]

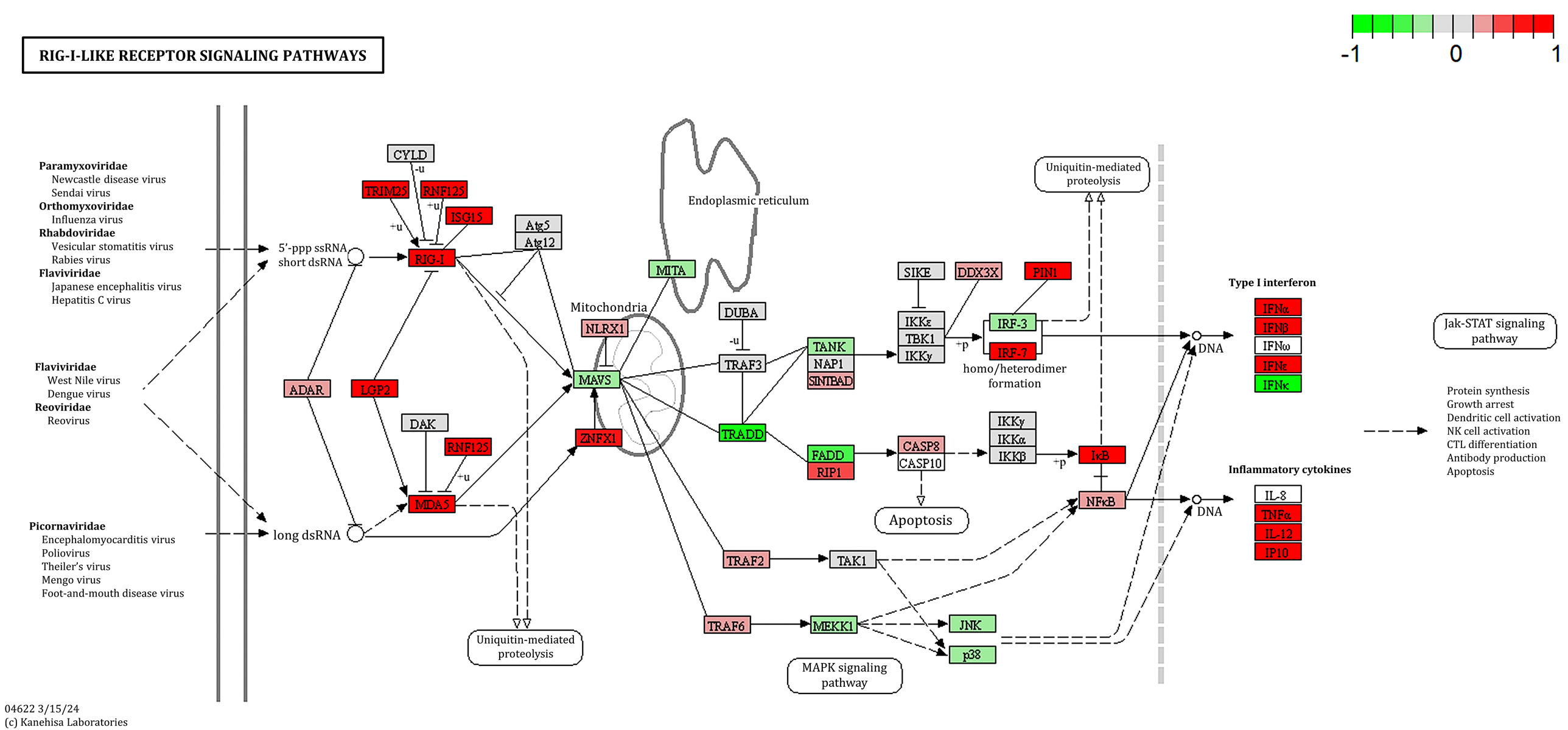

Supplement: Supplementary file 8 — Additional file 8. RIG−I-like receptor signaling pathway in PRE. [file 13567_2025_1650_MOESM8_ESM.jpg]
