# Supplementary material for: Transcriptomic landscape of pseudorabies virus-induced encephalitis reveals key lncRNAs involved in host–neurotropic virus interactions
Source: Vet Res. 2025 Nov 10;56:216. doi: 10.1186/s13567-025-01650-5 (PMC12604289; doi:10.1186/s13567-025-01650-5)
Supplement: Supplementary file 5 — Additional file 5. Top 20 upregulated and downregulated DEmRNAs. [file 13567_2025_1650_MOESM5_ESM.docx]

**Additional file 5: Top 20 upregulated and downregulated DEmRNAs in PRV-infected mouse brains.**

| **Gene_name** | **log_2_FoldChange** | ***p* value** | ***p*adj** | **Gene_type** | **Regulation** |
| --- | --- | --- | --- | --- | --- |
| Slc10a6 | 8.223038062 | 5.44E-14 | 3.51E-12 | protein_coding | Upregulated |
| Cxcl2 | 7.768263745 | 3.99E-12 | 2.06E-10 | protein_coding | Upregulated |
| Cxcr2 | 7.2118916 | 8.89E-10 | 3.16E-08 | protein_coding | Upregulated |
| Selp | 7.155419977 | 4.63E-10 | 1.74E-08 | protein_coding | Upregulated |
| Ms4a8a | 6.920950961 | 6.83E-08 | 1.68E-06 | protein_coding | Upregulated |
| Saa3 | 6.869451753 | 1.34E-26 | 2.22E-24 | protein_coding | Upregulated |
| Cxcl9 | 6.732256179 | 7.75E-09 | 2.36E-07 | protein_coding | Upregulated |
| Lcn2 | 6.677078612 | 2.76E-82 | 3.46E-79 | protein_coding | Upregulated |
| Cxcl10 | 6.501851373 | 6.97E-91 | 1.09E-87 | protein_coding | Upregulated |
| Il1rn | 6.275700034 | 3.17E-09 | 1.04E-07 | protein_coding | Upregulated |
| H2-Q6 | 6.172621869 | 3.38E-06 | 5.65E-05 | protein_coding | Upregulated |
| Slfn4 | 6.112694821 | 1.51E-12 | 8.22E-11 | protein_coding | Upregulated |
| Ms4a4c | 5.799322987 | 4.46E-22 | 5.80E-20 | protein_coding | Upregulated |
| Gm4841 | 5.663502081 | 2.40E-14 | 1.63E-12 | protein_coding | Upregulated |
| Ccl2 | 5.55008091 | 3.83E-45 | 1.37E-42 | protein_coding | Upregulated |
| Ifi205 | 5.421288136 | 1.75E-14 | 1.20E-12 | protein_coding | Upregulated |
| Mx1 | 5.390415084 | 1.84E-79 | 2.00E-76 | protein_coding | Upregulated |
| Trem1 | 5.37926489 | 3.18E-06 | 5.36E-05 | protein_coding | Upregulated |
| Zbp1 | 5.246580102 | 2.23E-39 | 6.65E-37 | protein_coding | Upregulated |
| Oas3 | 5.129688351 | 9.87E-19 | 1.05E-16 | protein_coding | Upregulated |
| Gkn3 | -4.219677134 | 2.48E-17 | 2.23E-15 | protein_coding | Downregulated |
| Or4d1 | -3.457218425 | 0.000835664 | 0.006536643 | protein_coding | Downregulated |
| Rpe65 | -3.423071834 | 6.02E-15 | 4.35E-13 | protein_coding | Downregulated |
| Abcg3 | -3.350941171 | 8.93E-06 | 0.000134002 | protein_coding | Downregulated |
| Or14j4 | -3.015927678 | 2.21E-06 | 3.84E-05 | protein_coding | Downregulated |
| Fam178b | -2.888656676 | 0.000959675 | 0.007297141 | protein_coding | Downregulated |
| Ly6g6f | -2.851208614 | 2.89E-08 | 7.82E-07 | protein_coding | Downregulated |
| Slc2a5 | -2.423522696 | 4.44E-14 | 2.93E-12 | protein_coding | Downregulated |
| Fabp7 | -2.414964399 | 7.10E-33 | 1.66E-30 | protein_coding | Downregulated |
| Myct1 | -2.37305705 | 0.000209087 | 0.002046126 | protein_coding | Downregulated |
| Gpr34 | -2.343465373 | 6.94E-48 | 2.95E-45 | protein_coding | Downregulated |
| Akr1c14 | -2.284248113 | 2.35E-16 | 1.98E-14 | protein_coding | Downregulated |
| Gdpd4 | -2.196518673 | 0.000450426 | 0.003895303 | protein_coding | Downregulated |
| Serpinb1a | -2.152757723 | 2.17E-36 | 5.90E-34 | protein_coding | Downregulated |
| AU021092 | -2.08447983 | 3.12E-10 | 1.21E-08 | protein_coding | Downregulated |
| Pbk | -2.076785436 | 6.86E-05 | 0.000787517 | protein_coding | Downregulated |
| Gm17455 | -2.069594633 | 0.003675486 | 0.021792057 | protein_coding | Downregulated |
| P2ry10b | -2.045303242 | 3.31E-05 | 0.000424035 | protein_coding | Downregulated |
| Slc38a5 | -1.949462102 | 3.04E-07 | 6.44E-06 | protein_coding | Downregulated |
| P2ry12 | -1.943834493 | 2.92E-51 | 1.33E-48 | protein_coding | Downregulated |
