# Supplementary material for: Transcriptomic landscape of pseudorabies virus-induced encephalitis reveals key lncRNAs involved in host–neurotropic virus interactions
Source: Vet Res. 2025 Nov 10;56:216. doi: 10.1186/s13567-025-01650-5 (PMC12604289; doi:10.1186/s13567-025-01650-5)
Supplement: Supplementary file 6 — Additional file 6. Top 20 upregulated and downregulated DElncRNAs. [file 13567_2025_1650_MOESM6_ESM.docx]

**Additional file 6: Top 20 upregulated and downregulated DElncRNA in PRV-infected mouse brains.**

| **Gene_name** | **log_2_FoldChange** | ***p* value** | ***p*adj** | **Gene_type** | **Regulation** |
| --- | --- | --- | --- | --- | --- |
| Gm29290 | 7.56295312 | 0.005895589 | 0.031754836 | lncRNA | Upregulated |
| Gm50237 | 6.508947837 | 6.24E-08 | 1.55E-06 | lncRNA | Upregulated |
| Gm35287 | 6.20304158 | 4.64E-07 | 9.43E-06 | lncRNA | Upregulated |
| 9330175E14Rik | 6.172827255 | 1.17E-07 | 2.73E-06 | lncRNA | Upregulated |
| Gm19951 | 4.534505056 | 0.000129751 | 0.001361731 | lncRNA | Upregulated |
| Gm36043 | 4.386056085 | 3.55E-09 | 1.15E-07 | lncRNA | Upregulated |
| Gm32568 | 4.290158787 | 0.000250565 | 0.002376857 | lncRNA | Upregulated |
| Gm11827 | 4.147041193 | 3.26E-05 | 0.000418506 | lncRNA | Upregulated |
| ENSMUSG00000121903 | 3.943292854 | 7.83E-10 | 2.82E-08 | lncRNA | Upregulated |
| Gm56710 | 3.312870203 | 0.001486636 | 0.010424754 | lncRNA | Upregulated |
| Gm49895 | 3.249339849 | 0.000591679 | 0.004903656 | lncRNA | Upregulated |
| Gm44850 | 3.190090827 | 7.41E-26 | 1.18E-23 | lncRNA | Upregulated |
| Gm40124 | 3.014155414 | 2.23E-08 | 6.16E-07 | lncRNA | Upregulated |
| Gm34648 | 2.997082788 | 0.003404982 | 0.020508449 | lncRNA | Upregulated |
| F730311O21Rik | 2.924652034 | 0.000390322 | 0.003446884 | lncRNA | Upregulated |
| BE692007 | 2.797885179 | 2.55E-09 | 8.46E-08 | lncRNA | Upregulated |
| Gm22146 | 2.751981532 | 2.38E-15 | 1.81E-13 | lncRNA | Upregulated |
| 4930512H18Rik | 2.751281109 | 0.001944559 | 0.012995876 | lncRNA | Upregulated |
| Gm38569 | 2.749139282 | 0.001305152 | 0.009391888 | lncRNA | Upregulated |
| Gm49838 | 2.590015436 | 3.04E-16 | 2.52E-14 | lncRNA | Upregulated |
| Gm46392 | -3.713961338 | 0.000173842 | 0.001750435 | lncRNA | Downregulated |
| Gm31115 | -2.744093441 | 0.000104753 | 0.001132575 | lncRNA | Downregulated |
| Gm12324 | -2.294361472 | 0.001919677 | 0.012846712 | lncRNA | Downregulated |
| C030029H02Rik | -2.237644117 | 4.59E-22 | 5.93E-20 | lncRNA | Downregulated |
| Gm14029 | -2.157769033 | 0.001712593 | 0.011685545 | lncRNA | Downregulated |
| A230001M10Rik | -2.139091231 | 3.48E-23 | 4.91E-21 | lncRNA | Downregulated |
| Gm28294 | -2.134235784 | 0.002557436 | 0.016201838 | lncRNA | Downregulated |
| AU023762 | -2.061320358 | 1.60E-09 | 5.49E-08 | lncRNA | Downregulated |
| Gm31135 | -2.058704212 | 0.001330173 | 0.009546619 | lncRNA | Downregulated |
| Gm48508 | -2.005986539 | 1.78E-05 | 0.000247926 | lncRNA | Downregulated |
| Gm10863 | -1.985817245 | 9.98E-08 | 2.37E-06 | lncRNA | Downregulated |
| Gm57389 | -1.970421819 | 2.79E-10 | 1.09E-08 | lncRNA | Downregulated |
| B230206H07Rik | -1.768811815 | 3.37E-06 | 5.65E-05 | lncRNA | Downregulated |
| Gm56823 | -1.741330417 | 0.003793484 | 0.022364688 | lncRNA | Downregulated |
| Gm13536 | -1.715684662 | 0.000725986 | 0.005826021 | lncRNA | Downregulated |
| 6530411M01Rik | -1.679243925 | 0.00034811 | 0.003133744 | lncRNA | Downregulated |
| C030005K06Rik | -1.67461362 | 0.00010217 | 0.001109439 | lncRNA | Downregulated |
| Gm57224 | -1.654737196 | 0.000863111 | 0.006707379 | lncRNA | Downregulated |
| Gm33727 | -1.648925789 | 0.008059488 | 0.040567762 | lncRNA | Downregulated |
| Gm36495 | -1.64809553 | 0.006241328 | 0.033266727 | lncRNA | Downregulated |
