# Supplementary material for: Transcriptomic landscape of pseudorabies virus-induced encephalitis reveals key lncRNAs involved in host–neurotropic virus interactions
Source: Vet Res. 2025 Nov 10;56:216. doi: 10.1186/s13567-025-01650-5 (PMC12604289; doi:10.1186/s13567-025-01650-5)
Supplement: Supplementary file 10 — Additional file 10. Detailed top correlations between lncRNAs and mRNAs. [file 13567_2025_1650_MOESM10_ESM.docx]

**Additional file 10: Detailed top correlation between lncRNAs and mRNAs.**

| **Source (lncRNA)** | **Target (mRNA)** | **Weight** |
| --- | --- | --- |
| Gm43660 | Tma16 | 0.998583 |
| B230206H07Rik | Bfsp2 | 0.997392 |
| Gm28294 | Or14j4 | 0.996773 |
| Gm57239 | Trim12a | 0.99674 |
| ENSMUSG00000121903 | Sult1a1 | 0.99664 |
| Zfas1 | Sh2b2 | 0.99663 |
| Zfas1 | Zc3hav1 | 0.995973 |
| Gm49838 | Saa3 | 0.995794 |
| Gm6209 | Ly6g6f | 0.995641 |
| Gm57191 | Potegl | 0.995511 |
| Zfas1 | Parp12 | 0.995505 |
| Gm20559 | Tlr2 | 0.995231 |
| Gm14029 | Tex52 | 0.995147 |
| Gm30489 | Slc2a4 | 0.995057 |
| B230206H07Rik | Itm2a | 0.994864 |
| Gm31135 | Gpc2 | 0.994463 |
| Gm44850 | Ccl2 | 0.994333 |
| Gm44850 | Oas1a | 0.994325 |
| Zfas1 | Ifi35 | 0.99419 |
| Gm10790 | Adgre1 | 0.99412 |
| Gm40124 | Gm5127 | 0.993876 |
| Gm21986 | Oas3 | 0.993798 |
| Gm56555 | Trib3 | 0.99378 |
| Gm57239 | Acer2 | 0.993638 |
| C030018K13Rik | Bfsp2 | 0.99348 |
| 4930512H18Rik | Muc1 | 0.993419 |
| Zfas1 | Atosa | 0.993333 |
| Zfas1 | Agt | 0.993182 |
| Zfas1 | Tgm2 | 0.993108 |
| Gm20559 | Tma16 | 0.993056 |
| Gm56917 | Nr4a3 | 0.993019 |
| C030018K13Rik | Tmprss5 | 0.992978 |
| Ppp1r36dn | Myh15 | 0.992921 |
| 2810407A14Rik | Gucy2g | 0.992904 |
| A230001M10Rik | Fabp7 | 0.992788 |
| Gm29290 | Gem | 0.99276 |
| AW112010 | Icam1 | 0.992651 |
| Gm57191 | Btla | 0.992503 |
| Gm45774 | C1ra | 0.992494 |
| BE692007 | Psmb8 | 0.992372 |
| G530011O06Rikx | Klrk1 | 0.992355 |
| E230001N04Rik | Epha2 | 0.9923 |
| C030018K13Rik | Steap3 | 0.992235 |
| Gm54109 | Fzd10 | 0.992132 |
| Neat1 | Slc45a3 | 0.992091 |
| Gm49838 | Ms4a6c | 0.992086 |
| Zfas1 | Ddc | 0.991916 |
| Pitpnm2os2 | Slc28a2 | 0.991823 |
| C030029H02Rik | Opalin | 0.991668 |
| Gm57239 | Mafk | 0.991651 |
| Gm57191 | Il6 | 0.991591 |
| Gm44850 | Csf3 | 0.991468 |
| Gm20559 | Pdk4 | 0.991464 |
| Gm13536 | Anpep | 0.991361 |
| G530011O06Rikx | Cxcr2 | 0.991267 |
| Gm45774 | Pla1a | 0.99111 |
| C330020E22Rik | Il12rb1 | 0.991064 |
| Gm56710 | Cd69 | 0.991025 |
| Neat1 | Clec4d | 0.990993 |
| Gm49895 | Klrk1 | 0.99096 |
| C030018K13Rik | Siglech | 0.990945 |
| Gm57239 | Fcgr3 | 0.990913 |
| Gm21986 | Pla2g3 | 0.990909 |
| ENSMUSG00000121903 | Patl2 | 0.990869 |
| Gm11266 | Gpr34 | 0.99081 |
| A230001M10Rik | Slc2a5 | 0.990769 |
| Zfas1 | Klf15 | 0.990672 |
| D17H6S56E-5 | Dnah17 | 0.990614 |
| Zfas1 | Spns2 | 0.990529 |
| Gm10030 | Apold1 | 0.990434 |
| C030018K13Rik | Klf10 | 0.990425 |
| 2810407A14Rik | C1qtnf2 | 0.990425 |
| Gm21986 | Arrdc2 | 0.990414 |
| Gm44421 | Gbp6 | 0.990318 |
| Gm45774 | Psmb8 | 0.990259 |
| Gm54109 | Egr3 | 0.990256 |
| ENSMUSG00000121903 | Otoa | 0.990143 |
| Gm40124 | 1700093K21Rik | 0.990111 |
| Med9os | Sh3rf2 | 0.990089 |
| E230001N04Rik | Gm12185 | 0.990088 |
| A230001M10Rik | Depdc7 | 0.990057 |
